# Role of Tunneling Nanotubes in Arachidonic Acid Transfer and Macrophage Function Reprogramming in Intrahepatic Cholangiocarcinoma

**DOI:** 10.1002/advs.202500148

**Published:** 2025-06-24

**Authors:** Meiru Chen, Shangyumeng Zhao, Xiaoli Xie, Jiaqi Wang, Miao Su, Lixian Zhang, Ruolin Cui, Dongqiang Zhao

**Affiliations:** ^1^ Department of Gastroenterology The Second Hospital of Hebei Medical University Hebei Key Laboratory of Gastroenterology Hebei Institute of Gastroenterology Hebei Clinical Research Center for Digestive Diseases Shijiazhuang Hebei 050000 China; ^2^ Department of Gastroenterology Hengshui People's Hospital Hengshui Hebei 053000 China; ^3^ Department of Preventive Medicine College of Public Health Hebei Medical University Shijiazhuang 050017 China

**Keywords:** arachidonic acid, intrahepatic cholangiocarcinoma, PI3K‐AKT signaling pathway, tumor‐associated macrophages, tunneling nanotubes

## Abstract

Tumor‐associated macrophages (TAMs) play a crucial role in tumor progression within the tumor microenvironment (TME) through phenotypic plasticity and functional modulation. While tunneling nanotubes (TNTs) mediate intercellular communication, their role in shaping TAMs phenotypes and function remains unclear. This study explores how TNTs facilitate the transfer of tumor‐derived materials, particularly fatty acids, to TAMs, affecting macrophage polarization and function. Single‐cell RNA sequencing identified heterogeneous macrophage subpopulations in the TME. Enrichment analysis pinpointed key substances transferred via TNTs. Lipidomics and metabolomics analyzed the fatty acids involved. In vitro and in vivo experiments validated TNTs‐mediated material transfer, and transcriptomic analysis revealed the associated signaling pathways. TNTs are the primary route for transferring tumor‐derived fatty acids, notably arachidonic acid (AA), to macrophages. This transfer reprogrammed TAMs from anti‐tumor CD5L^+^ to pro‐tumor TREM2^+^ phenotypes, increasing CCL18 secretion, reducing phagocytic activity, and impairing CD8^+^ T cell proliferation. Mechanistically, AA activated the PI3K‐AKT pathway, driving TAMs polarization. These findings are confirmed in xenograft models, where TNTs‐induced TAMs exhibited enhanced pro‐tumor properties. TNTs‐mediated transfer of tumor‐derived AA reprograms TAMs via PI3K‐AKT activation, promoting immune suppression and tumor progression, highlighting TNTs and PI3K‐AKT as potential therapeutic targets in iCCA.

## Graphical abstract

Tunneling nanotubes facilitate the transfer of long‐chain fatty acids, primarily arachidonic acid, from tumor cells to macrophages, leading to lipid accumulation and activation of the PI3K‐Akt pathway. This drives a shift from the anti‐tumor CD5L⁺ phenotype to a pro‐tumor TREM2⁺ phenotype. The transition reduces phagocytosis, impairs immune responses, and increases the release of the tumor‐promoting chemokine CCL18, reprogramming macrophage function and creating an immunosuppressive microenvironment.

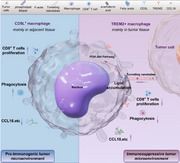

## 1 Introduction

Intrahepatic cholangiocarcinoma (iCCA) is a highly aggressive malignancy with limited therapeutic options and dismal overall survival rates.^[^ ^1^ ^]^ The progression of iCCA is closely linked to the tumor microenvironment (TME), where tumor‐associated macrophages (TAMs) play a central role in promoting immunosuppression, angiogenesis, and tumor proliferation. These processes significantly enhance the aggressiveness of iCCA, making TAMs attractive targets for immunotherapeutic interventions.^[^ ^2^ ^]^ While tumor‐driven polarization of TAMs toward immunosuppressive, tumor‐supportive phenotypes is well established, the precise mechanisms driving this reprogramming.^[^ ^3^ ^]^ A deeper understanding of how iCCA tumor cells influence TAMs phenotypes could uncover novel therapeutic targets and advance cancer immunotherapy strategies.

Emerging evidence highlights the importance of tumor‐derived factors in TAMs reprogramming.^[^ ^4^ ^]^ Among these, arachidonic acid (AA) and its derivatives have been implicated in driving TAMs toward an M2‐like, pro‐tumor phenotype in several cancers.^[^ ^5^ ^]^ Despite these findings, the mechanisms through which AA and other tumor‐derived metabolites influence macrophage polarization, particularly in iCCA, remain elusive. Exploring these mechanisms is crucial to decipher the metabolic and functional interplay between tumor cells and TAMs within the TME.

Intercellular communication is fundamental to TME remodeling and tumor progression. While extracellular vesicles, cytokines, and chemokines have been extensively studied,^[^ ^4^, ^6^ ^]^ recent research has identified tunneling nanotubes (TNTs) as a novel mode of direct intercellular communication.^[^ ^7^ ^]^ TNTs are thin, actin‐based structures that facilitate the transfer of diverse cellular contents, including proteins, organelles, and metabolites, across distant cells.^[^ ^8^ ^]^ Notably, TNTs have been shown to enhance cancer cell metabolic plasticity, invasiveness, immune evasion, and resistance to therapy.^[^ ^9^, ^10^, ^11^ ^]^ However, their specific role in regulating macrophage polarization within the TME remains largely unexplored. Given the critical involvement of TAMs in tumor progression, elucidating how tumor cells influence TAMs function via TNTs may uncover novel mechanisms driving TME remodeling and tumor progression.

In this study, we explored the role of TNTs in facilitating the transfer of tumor‐derived fatty acids, particularly AA, to TAMs within the iCCA microenvironment. Through integrated analyses involving single‐cell RNA sequencing (scRNA‐seq), lipidomics, targeted metabolomics, and transcriptomics, AA was identified as the predominant fatty acid transferred via TNTs. Mechanistically, AA was shown to activate the PI3K‐Akt signaling pathway, reprogramming TAMs into a pro‐tumorigenic TREM2⁺ phenotype marked by diminished phagocytic capacity and impaired support for CD8⁺ T cell proliferation. These findings offer novel insights into TNTs‐mediated tumor‐driven remodeling of the TME and highlight potential therapeutic targets for cancer treatment.

## 2 Results

### 2.1 scRNA‐Seq Analysis and In Vitro Experiments Reveal the Advantage of Tumor Cells in TNT Formation

**Figure 1:**
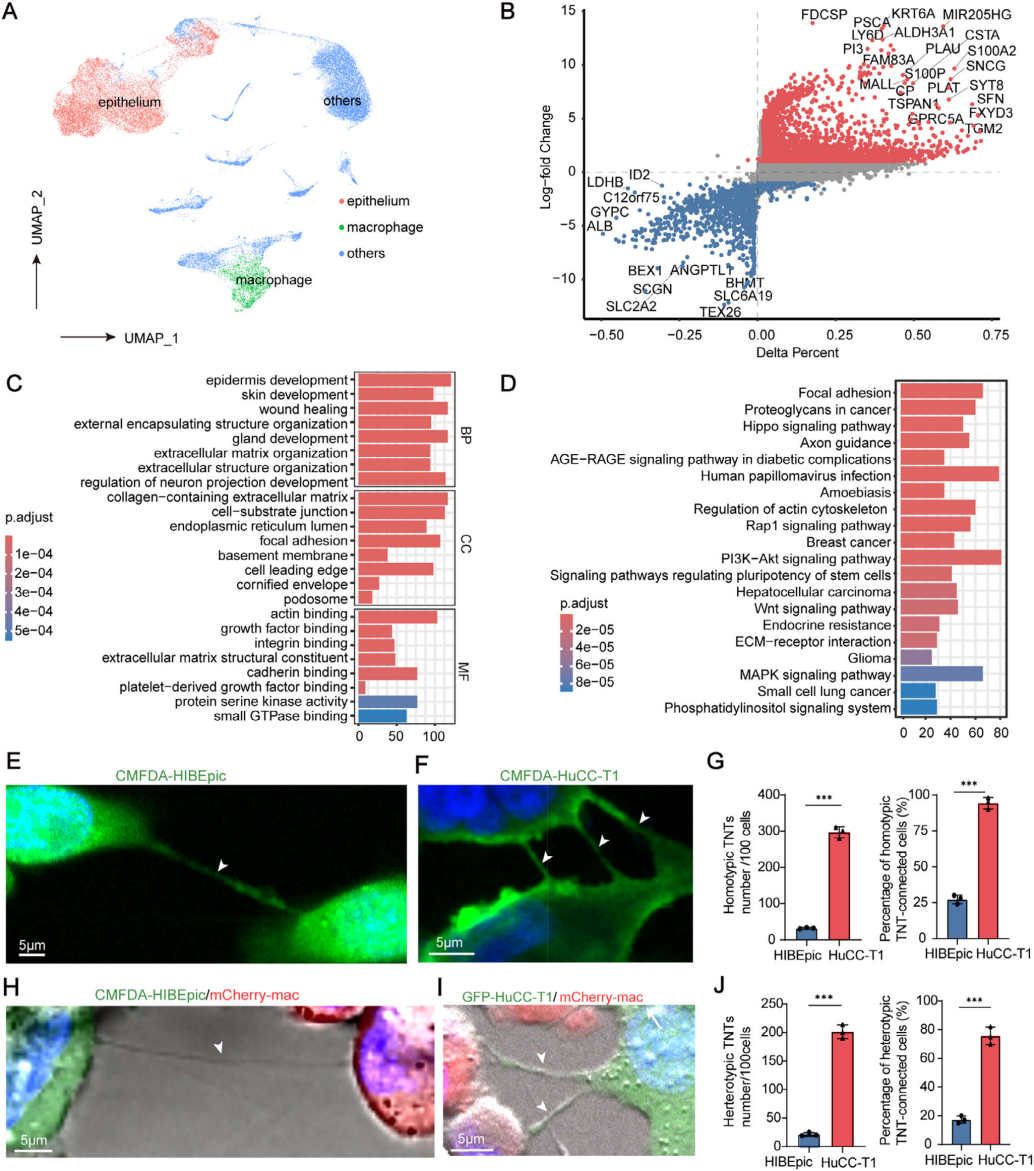
scRNA‐seq analysis and in vitro experiments reveal tumor cell advantage in TNT formation. A) UMAP plot of epithelial cells and macrophages. B) Volcano plot showing differentially expressed genes between tumor and adjacent non‐tumor epithelial cells. C,D) GO and KEGG pathway enrichment analyses of upregulated genes in tumor cells. E,F) Representative images of homotypic TNTs formed between CMFDA‐labeled HIBEpic cells (E) and CMFDA‐HuCC‐T1 cells (F). White arrows indicate TNTs. G) Quantification of homotypic TNT connections per 100 cells (left) and percentage of TNT‐connected cells (right) between HIBEpic and HuCC‐T1. Data are presented as mean ± SD; n = 3. H,I) Heterotypic TNTs between CMFDA‐HIBEpic (H) or GFP‐HuCC‐T1 (I) and mCherry‐labeled macrophages. J) Quantification of heterotypic TNT connections per 100 cells (left) and percentage of TNT‐connected cells (right) between HIBEpic/HuCC‐T1 and macrophages. Data are presented as mean ± SD; n = 3.

To investigate the role of tumor cells in initiating TNTs formation, we performed an in‐depth analysis of the genetic alterations in epithelial cells within tumor tissues to uncover their potential association with TNTs formation. ScRNA‐seq data analysis identified 23 distinct cell clusters (Figure S1A, Supporting Information), and cell types were classified based on marker gene expression (Figure S1B, Supporting Information), successfully distinguishing cholangiocytes from macrophages (**Figure** **1**A). Differential expression analysis further revealed significant differences in gene expression between tumor epithelial cells and adjacent normal epithelial cells (Figure 1B).

GO functional analysis of the upregulated genes in tumor cells demonstrated significant enrichment in processes such as focal adhesion, actin binding, extracellular matrix organization, and small GTPase binding (Figure 1C). Additionally, KEGG pathway analysis revealed that these genes were mainly involved in focal adhesion, regulation of the actin cytoskeleton, the PI3K‐AKT signaling pathway, and the MAPK signaling pathway (Figure 1D). These biological processes and pathways are well‐documented for their pivotal role in the formation of TNTs, as extensively studied in the literature.^[^ ^12^, ^13^, ^14^ ^]^ These results suggest that tumor cells in iCCA possess a strong intrinsic capacity to initiate and facilitate TNTs formation, potentially through the activation of actin cytoskeleton‐associated pathways and signaling cascades involved in cellular adhesion and motility.

Additionally, through in vitro experiments, we demonstrated that tumor cells exhibit a superior capacity for TNT formation compared to normal bile duct epithelial cells. Specifically, the percentage and number of homotypic TNTs formed between normal human intrahepatic biliary epithelial cells (HIBEpiC) were significantly lower than those observed among HuCC‐T1 tumor cells (Figure 1E–G). Likewise, heterotypic TNTs between HIBEpiC cells and macrophages were substantially less frequent compared to those between HuCC‐T1 tumor cells and macrophages (Figure 1H–J). These differences were statistically significant (*p* < 0.001), providing both quantitative data and visual evidence of TNT formation across these cell types. This direct observation not only corroborates the molecular insights derived from scRNA‐seq but also offers compelling functional evidence of the enhanced TNT formation capacity of iCCA tumor cells.

### 2.2 Identification of Macrophage Phenotypes and Cytokines Associated with THEIR function

**Figure 2:**
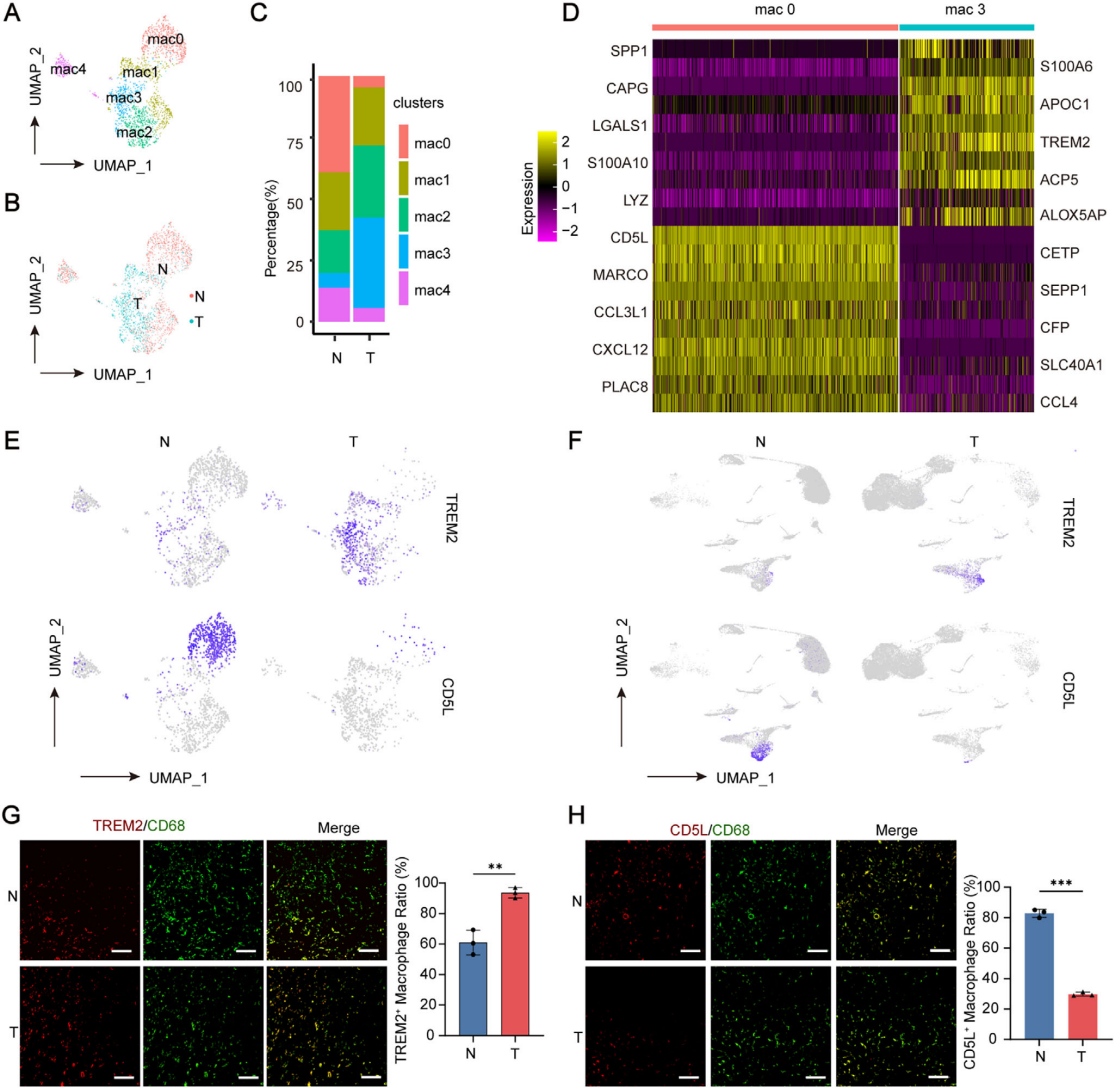
Identification of macrophage subtypes and associated functional markers. A) UMAP plot of five macrophage subtypes. B) Distribution of macrophage subtypes in tumor (T) and normal (N) tissues. C) Proportions of macrophage subtypes in tumor versus normal tissues. D) Heatmap of top 10 differentially expressed genes between mac0 and mac3. E) UMAP visualization of TREM2 (top) and CD5L (bottom) expression across macrophage subtypes in tumor and normal tissues. F) The expression levels of TREM2 (top) and CD5L (bottom) across cell types in tumor and normal tissues. G,H) Quantitative analysis of TREM2 (G) and CD5L (H) expression in macrophages. Data are presented as mean ± SD; n = 3 (^**^ *p* < 0.01, ^***^ *p* < 0.001). Scale bar: 100 µm.

To elucidate the mechanisms through which tumor cells reshape the TME, we focused on macrophages, identified as the most heterogeneous immune cell population in our study.^[^ ^15^ ^]^ ScRNA‐seq analysis identified five distinct macrophage subtypes (**Figure** **2**A), which showed significant differences in their relative abundance between tumor tissues and adjacent normal tissues (Figure 2B,C). Specifically, the mac0 subtype was mainly enriched in normal tissues, whereas the mac3 subtype was significantly more abundant in tumor tissues. We isolated the mac0 and mac3 macrophage subtypes, excluding tumor cells from the mac0 group and normal cells from the mac3 group. The top 10 marker genes for both the mac0 and mac3 subtypes are shown in Figure 2D.

Consistent with prior studies, we observed that TREM2, a well‐known marker of immunosuppressive macrophages in multiple cancers^[^ ^16^ ^]^ was highly expressed in the mac3 subtype within tumor tissues. Conversely, CD5L, previously recognized for its anti‐tumor properties and positive correlation with CD8⁺ T cell infiltration and M1 macrophage activity^[^ ^17^ ^]^ was predominantly expressed in the mac0 subtype in normal tissues (Figure 2E). Across all analyzed cell types, TREM2 expression was primarily confined to tumor tissues, whereas CD5L was more prevalent in normal tissues (Figure 2F).

To validate these findings, multiple immunofluorescence (MIF) confirmed that TREM2⁺ macrophages were predominantly localized within iCCA tumor tissues (Figure 2G), while CD5L⁺ macrophages were the predominant subtype in peritumoral tissues (Figure 2H). Based on these results, we classified mac0 as “tumor‐suppressive CD5L⁺ macrophages” and mac3 as “tumor‐promoting TREM2⁺ macrophages”.

In addition, we identified CCL18, a member of the CC chemokine family primarily secreted by monocytes, as a potential driver of tumor progression. Previous studies have established its pivotal role in tumorigenesis across various cancers.^[^ ^18^, ^19^ ^]^ To further validate the pro‐tumorigenic role of CCL18, we conducted experiments by treating tumor cells with recombinant CCL18 (rCCL18). Using Cell counting kit‐8 (CCK‐8) cell proliferation and Transwell migration assays, we found that rCCL18 significantly enhanced the proliferative and migratory capabilities of tumor cells compared to the control group (Figure S1C,D, Supporting Information). Notably, our scRNA‐seq data revealed that CCL18 expression was mainly enriched in macrophages and significantly upregulated in the TREM2⁺ macrophage subset (Figure S1E,F, Supporting Information). This observation suggests that TREM2⁺ macrophages may contribute to tumor progression by secreting CCL18 and promoting pro‐tumorigenic effects within the TME.

### 2.3 The Presence of TNTs Between Tumor Cells and Macrophages

Building on our previous findings, we observed that differentially expressed genes in tumor cells and macrophages were significantly enriched in pathways associated with TNTs formation.^[^ ^15^ ^]^ Enrichment analyses further suggested that tumor cells possess an intrinsic ability to initiate TNTs formation. These observations led us to hypothesize that TNTs might serve as a structural link facilitating direct communication between tumor cells and macrophages.

**Figure 3:**
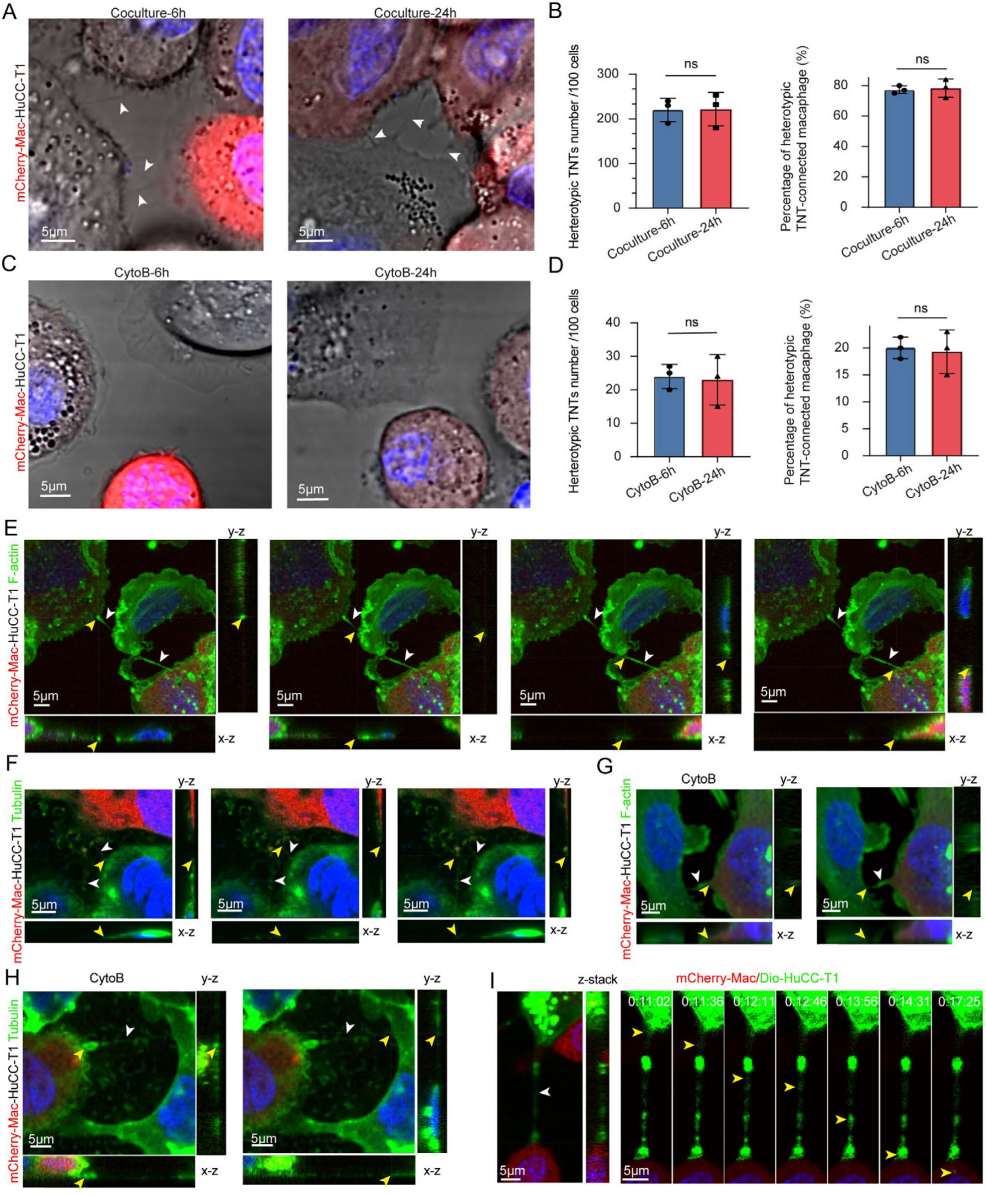
TNT formation dynamics between tumor cells and macrophages. A) TNTs (white arrows) formed between mCherry‐macrophages and HuCC‐T1 cells at 6 and 24 h. B) Quantification of TNT numbers (left) and percentage of TNT‐connected cells (right) at these time points. Data are mean ± SD; n = 3 (ns). C) Suppressed TNT formation after CytoB treatment at 6 and 24 h. D) Quantification of TNT numbers (left) and percentage of TNT‐connected cells (right) at these time points after CytoB treatment at these time points. Data are mean ± SD; n = 3 (ns). E,F) Immunofluorescence staining of F‐actin (phalloidin) and tubulin in TNTs. White arrows indicate TNTs, yellow arrows indicate orthogonal views. G,H) Disruption of cytoskeletal components by CytoB, immunofluorescence staining of F‐actin (phalloidin) and tubulin in TNTs. White arrows indicate TNTs, yellow arrows indicate orthogonal views. I) Transfer of DIO dye from HuCC‐T1 cells to macrophages via TNTs (white arrows). ns: not significant.

To test this hypothesis, we established a co‐culture system of tumor cells and macrophages and employed confocal microscopy to examine TNT formation between these cell types (Video S1, Supporting Information). Observations at 6 and 24 h showed no significant changes in the number or percentage of TNTs formed (**Figure** **3**A,B). Treatment with CytoB, a known inhibitor of actin polymerization, substantially suppressed TNT formation; however, the number and percentage of TNTs remained consistent between the 6 and 24‐h time points (Figure 3C,D).

The formation of TNTs between tumor cells and macrophages was a frequent and reproducible phenomenon within the co‐culture system. Structural analysis confirmed that these TNTs were primarily composed of F‐actin (Figure 3E) and microtubules (Figure 3F), with *Z*‐axis imaging demonstrating their lack of attachment to the basal substrate, consistent with established TNT characteristics.^[^ ^20^ ^]^ Although CytoB treatment markedly reduced the number and percentage of TNTs, the remaining TNTs retained the culture system. Structural analysis confirmed that these TNTs were primarily composed of F‐actin (Figure 3G) and microtubules (Figure 3H).

To confirm the functional role of these TNTs and distinguish them from other cellular protrusions,^[^ ^21^ ^]^we assessed their capacity for intercellular cargo transfer. Using the lipophilic dye DiO as a marker, we observed efficient transport of DiO‐labeled cargo from tumor cells to macrophages via TNTs (Figure 3I, Video S2, Supporting Information). These findings provide compelling evidence that TNTs not only establish physical connections between tumor cells and macrophages but also mediate the transfer of cellular materials, highlighting their critical role in facilitating tumor‐macrophage communication.

### 2.4 Effects of TNTs‐Mediated Material Transfer On Macrophage Phenotypes

In the TME, TAMs exhibit significant phenotypic plasticity, polarizing into either pro‐inflammatory M1‐like macrophages or pro‐tumorigenic M2‐like macrophages.^[^ ^22^ ^]^ Based on our findings, we hypothesized that TNTs‐mediated material transfer could drive macrophages toward a tumor‐promoting TREM2⁺ phenotype, a subtype associated with immunosuppression and tumor promotion.

To test this hypothesis, we employed flow cytometry to analyze the expression of key phenotypic markers TREM2 and CD5L in macrophages under different experimental conditions. These conditions included M0 (control macrophages), Coculture (macrophages co‐cultured with tumor cells), CytoB (TNTs formation inhibited by cytochalasin B), and Transwell (physical separation of macrophages and tumor cells) groups. Experiments were conducted using two cholangiocarcinoma cell lines, HuCC‐T1 and RBE. Additionally, we quantified the secretion of the pro‐tumorigenic chemokine CCL18 in culture supernatants using enzyme‐linked immunosorbent assay (ELISA).

**Figure 4:**
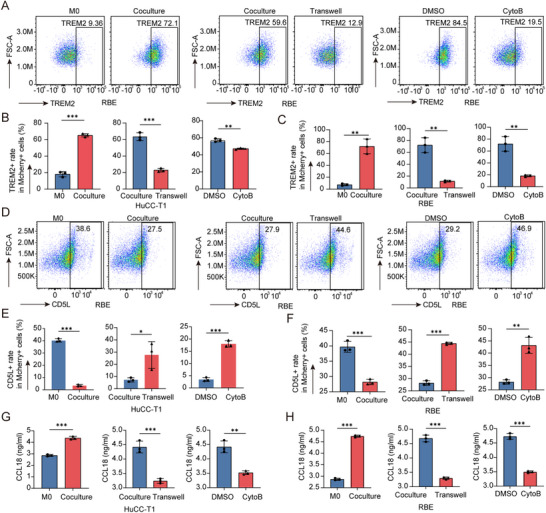
TNT‐mediated material transfer modulates macrophage phenotypes. A) Flow cytometry analysis of TREM2 expression in macrophages co‐cultured with RBE cells. B,C) Quantification of TREM2^+^ macrophages in HuCC‐T1 (B) and RBE (C) co‐culture systems. D) Flow cytometry analysis of CD5L expression. (E‐F) Quantification of CD5L^+^ macrophages in HuCC‐T1 E) and RBE F) systems. G,H) CCL18 secretion levels in HuCC‐T1 (G) and RBE (H) systems. Data are mean ± SD; n = 3 (^*^ *p* < 0.05, ^**^ *p* < 0.01, ^***^ *p* < 0.001).

The results demonstrated a significant increase in TREM2⁺ macrophages in the Coculture group compared to other groups (**Figure** **4**A–C; Figure S2A, Supporting Information), alongside a marked reduction in CD5L expression (Figure 4D–F; Figure S2B, Supporting Information). Furthermore, macrophages in the Coculture group exhibited substantially elevated levels of CCL18 secretion (Figure 4G,H), indicating a functional shift toward a tumor‐promoting phenotype. In contrast, TNTs inhibition (CytoB group) and physical separation (Transwell group) effectively suppressed these changes, supporting the role of TNTs‐mediated material transfer in driving macrophage phenotypic conversion.

These findings highlight the critical role of TNTs in facilitating the polarization of macrophages toward the TREM2⁺ phenotype and promoting the secretion of CCL18, a chemokine implicated in tumor progression. This underscores the functional significance of TNTs‐mediated communication between tumor cells and macrophages in reshaping macrophage behavior within the TME, ultimately contributing to an immunosuppressive and tumor‐supportive microenvironment.

### 2.5 Effects of TNTs‐Mediated Material Transfer on Macrophage Function

Tumor‐infiltrating immune cells, including CD8⁺ T cells and macrophages, are critical components of the tumor immune microenvironment (TIME). Among them, CD8⁺ T cells play a pivotal role in anti‐tumor immunity by mounting effective immune responses against tumors.^[^ ^23^ ^]^ However, immunosuppressive cells such as TAMs and myeloid‐derived suppressor cells often suppress CD8⁺ T cell function, thereby facilitating tumor progression in various cancers, including hepatocellular carcinoma.^[^ ^24^ ^]^ To investigate the effects of TNTs‐mediated material transfer on macrophage function, we evaluated three key parameters: macrophage phagocytic activity, their regulatory effects on CD8⁺ T cell proliferation, and their pro‐tumorigenic capacity.

**Figure 5:**
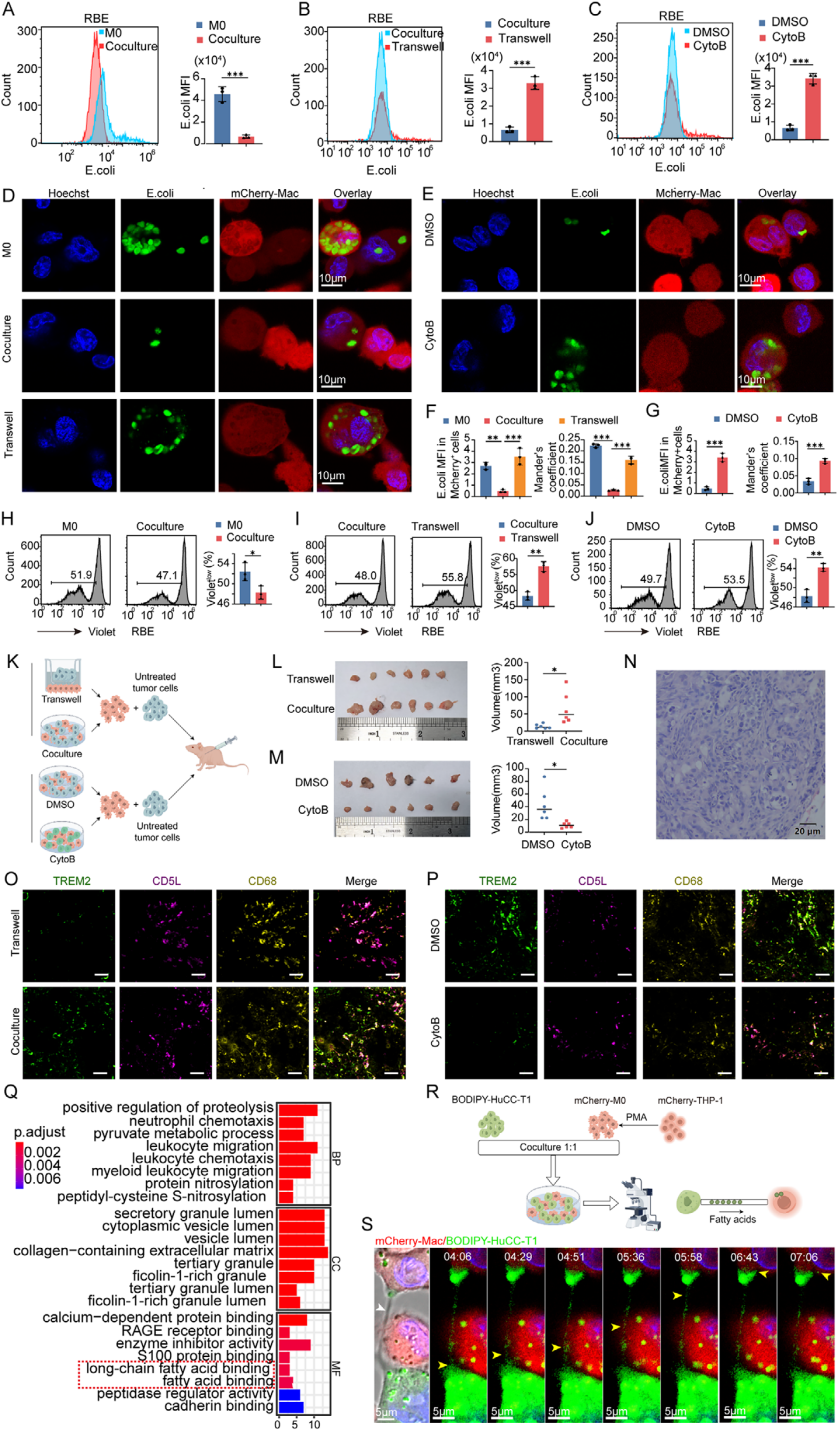
Functional consequences of TNT‐mediated material transfer. A–C) Flow cytometry analysis of *E. coli* phagocytic activity comparing the Coculture group with M0, Transwell, and CytoB groups, respectively, along with statistical comparison. Results are shown for the RBE coculture system. Data are mean ± SD; n = 3. D,E) Laser confocal microscopy showing macrophage phagocytosis of *E. coli*: M0, Coculture, and Transwell groups (D), and DMSO and CytoB groups (E).F,G) MFI and Mander's coefficient analysis of phagocytosis. Data are mean ± SD; n = 3. H–J) Proliferation of CD8^+^ T cells co‐cultured with macrophages. Results are shown for the RBE coculture system. Data are mean ± SD; n = 3. K) Schematic of xenograft experiments. L,M) Tumor volume comparisons. Data are mean ± SD; n = 6. N) H&E staining of tumors. (O‐P) Expression of TREM2 and CD5L in macrophages within xenograft tumors from the Coculture and Transwell groups O), and from the DMSO and CytoB groups P). Scale bar: 200 µm. Q) The molecular function of TREM2^+^‐macrophages was significantly enriched in fatty acid binding (red box). R) Flowchart of co‐culturing process to observe fatty acids transfer. (by Figdraw) S) Transfer of fatty acids from tumor cells to macrophages through TNTs. (Left) Bright‐field image of hucc‐t1 cells labeled with BODIPY 493/503 (green) co‐cultured with mCherry‐expressing macrophages (red). White arrows indicate TNTs bridging tumor cells and macrophages. (Right) Time‐lapse fluorescence imaging (*t*‐axis) reveals dynamic lipid trafficking through TNTs. Yellow arrows highlight BODIPY‐labeled fatty acid cargos transferred from tumor cells to macrophages over time. MFI, mean fluorescence intensity Significance is indicated as follows: ^*^, *p* < 0.05; ^**^, *p* < 0.01; ^***^, *p* < 0.001.

Flow cytometric analysis revealed that CytoB treatment, which inhibits TNTs formation, did not significantly affect macrophage phagocytic activity (Figure S3A, Supporting Information). However, in the coculture groups with HuCC‐T1 and RBE cells, the phagocytic activity of macrophages against *E. coli* was significantly reduced compared to the control groups, as determined by flow cytometry analysis (*p* < 0.05, **Figure** **5**A–C for RBE; Figure S3B–D for HuCC‐T1, Supporting Information). This reduction was further corroborated by confocal microscopy through the measurement of mean fluorescence intensity (MFI) and Mander's coefficient (*p* < 0.05, Figure 5D–G). Moreover, the phagocytosis of tumor cells provided additional evidence supporting this observation (Figure S3E–G, Supporting Information). These findings suggest that direct intercellular communication via TNTs impairs the functional phagocytic capacity of macrophages. Furthermore, when assessing the regulatory effects of macrophages on CD8⁺ T cell proliferation, we observed that proliferation was markedly suppressed in the Coculture groups compared to other experimental conditions (*p* < 0.05, Figure 5H–J for RBE; Figure S3H–J for HuCC‐T1, Supporting Information).

To evaluate the pro‐tumorigenic capacity of macrophages, we conducted in vivo experiments using a nude mouse xenograft model (workflow shown in Figure 5K). Tumor volumes were significantly larger in the Coculture group compared to the Transwell group (Figure 5L), and in the DMSO group compared to the CytoB group (Figure 5M), highlighting the role of TNTs formation in enhancing the pro‐tumorigenic potential of macrophages. Hematoxylin and eosin (H&E) staining further confirmed the heterogeneity of the xenografts (Figure 5N).

Immunofluorescence analysis revealed that macrophages in the Coculture group exhibited an increased proportion of TREM2^+^ macrophages and a decreased proportion of CD5L^+^ macrophages compared to the Transwell group (Figure 5O). Conversely, inhibition of TNTs formation by CytoB resulted in a lower proportion of TREM2^+^ macrophages and a higher proportion of CD5L^+^ macrophages compared to the DMSO group (Figure 5P).

In summary, TNTs‐mediated material transfer facilitates the polarization of macrophages toward a TREM2⁺ pro‐tumorigenic phenotype, diminishes their phagocytic activity, and suppresses CD8⁺ T cell proliferation. This process not only reinforces the tumor‐promoting role of macrophages but also impairs antitumor immune responses, contributing significantly to tumor progression in the iCCA microenvironment.

### 2.6 Tumor Cells Transfer Fatty Acids to Macrophages via TNTs

To identify the materials transferred from tumor cells to macrophages, we performed enrichment analysis on scRNA‐seq data from TREM2⁺ macrophages and CD5L⁺ macrophages. The analysis revealed that genes upregulated in the TREM2⁺ macrophage subset were significantly enriched in pathways related to fatty acid binding and long‐chain fatty acid binding (Figure 5Q). These findings align with previous studies demonstrating that lipids and their metabolic derivatives are key regulators of macrophage polarization, pro‐inflammatory and anti‐inflammatory properties, as well as phagocytic function.^[^ ^2^ ^]^ Based on this evidence, we hypothesized that tumor cells transfer fatty acids to macrophages via TNTs, resulting in lipid accumulation and driving their phenotypic transformation.

To test this hypothesis, we established an experimental workflow (Figure 5R). Using fluorescent labeling and advanced imaging techniques, we demonstrated that fatty acids could be efficiently transferred from tumor cells to macrophages through TNTs (Figure 5S, Video S3, Supporting Information). These findings provide novel mechanistic insights into how TNTs facilitate intercellular lipid transfer, contributing to macrophage phenotypic reprogramming and functional modulation within TME.

### 2.7 TNTs are the Primary Pathway for Tumor‐Derived Fatty Acid Transfer to Macrophages

To identify the primary mechanism by which tumor‐derived fatty acids are transferred to macrophages, we evaluated three potential pathways: (1) direct transfer via TNTs, (2) macrophage phagocytosis of tumor cells, and (3) uptake of tumor‐secreted fatty acids through macrophage CD36 scavenger receptors.^[^ ^25^, ^26^ ^]^

Using live‐cell confocal microscopy over a 13‐h observation period, no evidence of macrophages phagocytosing whole tumor cells was detected, ruling out phagocytosis as the primary pathway for fatty acid transfer (Video S4, Supporting Information). Additionally, scRNA‐seq analysis showed no significant upregulation of CD36 expression in macrophages within tumor samples, further diminishing the likelihood of CD36‐mediated uptake as the primary mechanism (Figure S4A, Supporting Information).

Tumor‐secreted vesicles have been previously reported to transport fatty acids to dendritic cells and TAMs.^[^ ^27^ ^]^ These vesicles, with diameters ranging from 50 to 500 nm, can freely pass through Transwell membranes with 1 µm pores.^[^ ^28^ ^]^ To investigate the potential role of vesicle‐mediated fatty acid transfer, we employed a Transwell system. Measurements of free fatty acid concentrations in the upper chambers of Coculture and Transwell systems revealed no significant differences (*p* > 0.05, Figure S4B,C, Supporting Information), indicating that the presence of the Transwell membrane did not impair fatty acid availability in the macrophage culture system.

**Figure 6:**
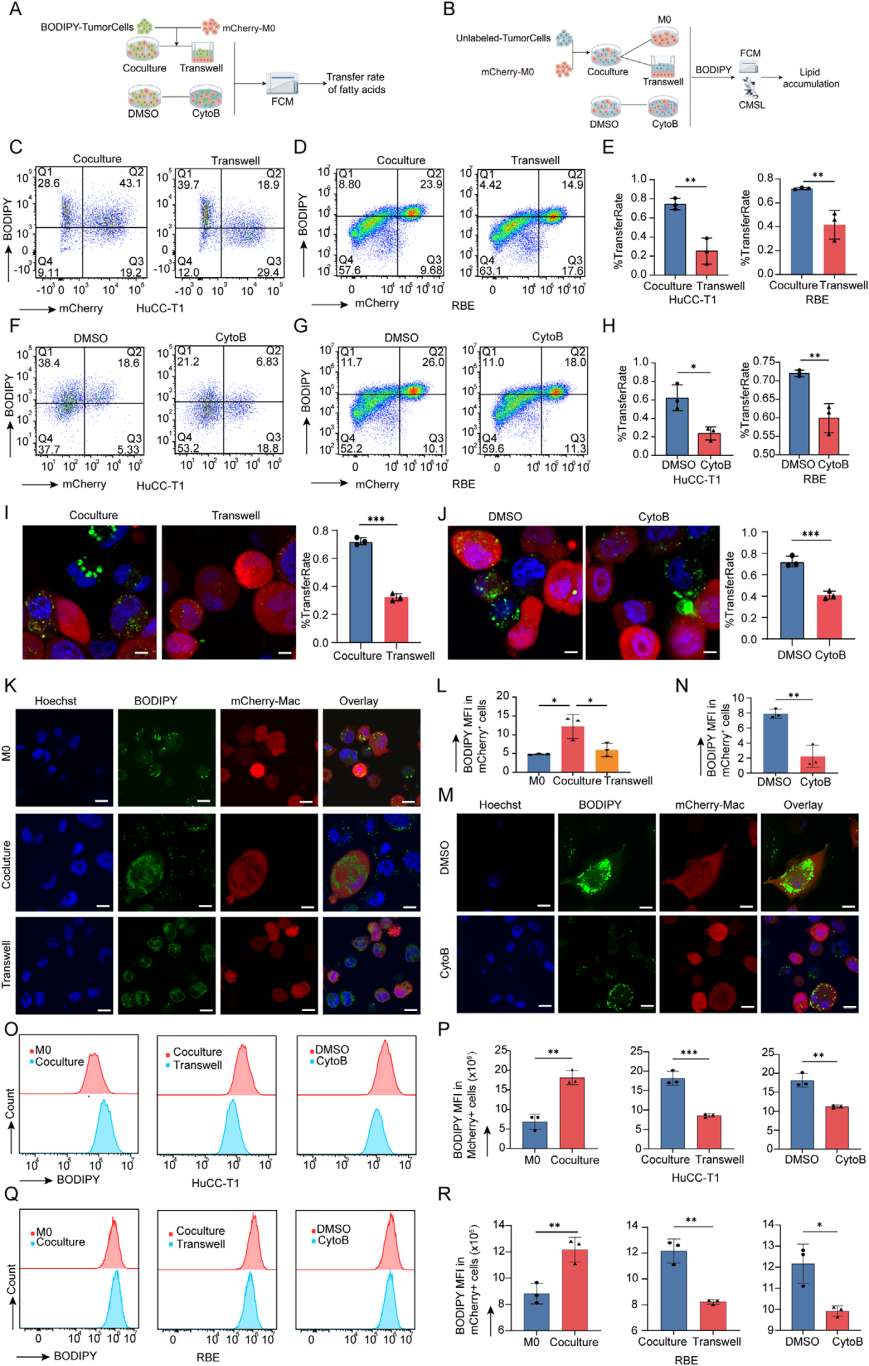
TNTs facilitate tumor‐derived fatty acid transfer to macrophages. A,B) Schematic workflows for quantifying fatty acid transfer efficiency (A) and macrophage lipid accumulation (B), designed using Figdraw. C–E) Comparative fatty acid transfer rates between Coculture and Transwell systems in HuCC‐T1 (C) and RBE (D) tumor cells, with pooled statistical analysis (E). F–H) Pharmacological inhibition of TNT‐mediated transfer using CytoB versus DMSO control in HuCC‐T1 (F) and RBE (G) models, summarized in panel H. Data represent mean ± SD; n = 3. I,J) Fluorescence microscopy quantification of BODIPY 493/503^+^ macrophages: Coculture systems exhibited enhanced fatty acid transfer compared to Transwell controls (I), while CytoB treatment significantly attenuated lipid trafficking (J) (mean ± SEM; n = 3). K–N) Confocal microscopy visualization of lipid accumulation: M0 macrophages (negative control), Coculture, and Transwell groups (K‐L), and DMSO/CytoB comparisons (M‐N). (mean ± SEM; n = 3) (O‐R) Flow cytometric validation of lipid accumulation in macrophages: HuCC‐T1 (O‐P) and RBE (Q‐R) coculture systems, demonstrating TNT‐dependent lipid transfer (mean ± SEM; n = 3). Significance levels: ^*^ *p* < 0.05, ^**^ *p* < 0.01, ^***^ *p* < 0.001. Scale bar, 10 µm.

To evaluate the role of TNTs in fatty acid transfer, we analyzed fatty acid transfer rates and lipid accumulation in macrophages, with the experimental workflow shown in **Figure** **6**A,B. Flow cytometry results demonstrated that fatty acid transfer rates in the Coculture group were significantly higher than those in the Transwell group (*p* < 0.05, Figure 6C–E). Moreover, treatment with the actin polymerization inhibitor CytoB significantly reduced fatty acid transfer rates (*p* < 0.05, Figure 6F–H), further supporting the critical role of TNTs in this process. To further validate these findings, we quantified the percentage of acceptor macrophages receiving fatty acid using fluorescence microscopy with BODIPY 493/503 staining. The Coculture group exhibited a significantly higher percentage of macrophages with transferred fatty acids compared to the Transwell group (*p* < 0.05, Figure 6I), consistent with the flow cytometry data. Similarly, the percentage was higher in the DMSO control group than in the CytoB‐treated group (*p* < 0.05, Figure 6J), providing additional evidence that TNTs facilitate fatty acid transfer.

Lipid accumulation in macrophages was further evaluated using BODIPY 493/503 staining, which specifically labels neutral lipids. In the Transwell group and macrophages before coculture, green fluorescent puncta were small and evenly distributed. However, macrophages in the Coculture group exhibited a marked increase in lipid droplets, observed as larger green fluorescent puncta (Figure 6K,L). Similar lipid accumulation patterns were observed in the DMSO group (Figure 6M,N). Flow cytometric analysis confirmed a significant increase in the MFI of BODIPY staining in macrophages from the Coculture group compared to controls, consistent across both HuCC‐T1 (*p* < 0.05, Figure 6O,P) and RBE cells (*p* < 0.05, Figure 6Q,R).

In conclusion, our findings unequivocally demonstrate that TNTs serve as the primary pathway for the transfer of fatty acids from tumor cells to macrophages. The significantly higher fatty acid transfer rates and lipid accumulation observed in the Coculture group underscore the dominant role of TNTs in this process.

### 2.8 TNTs‐Mediated AA Transfer Drives Macrophage Phenotypic Transformation and Functional Reprogramming

Metabolic crosstalk between tumor cells and macrophages plays a critical role in shaping the immunosuppressive TME.^[^ ^29^ ^]^ M2‐like TAMs are considered the result of tumor cell reprogramming, with previous studies highlighting the importance of AA metabolites in regulating M2‐TAMs formation and promoting tumor progression.^[^ ^30^ ^]^ In this study, we investigated the types of fatty acids transferred via TNTs and their impact on macrophage phenotype and function using lipidomics, oxidized lipid analysis, and targeted metabolic profiling.

**Figure 7:**
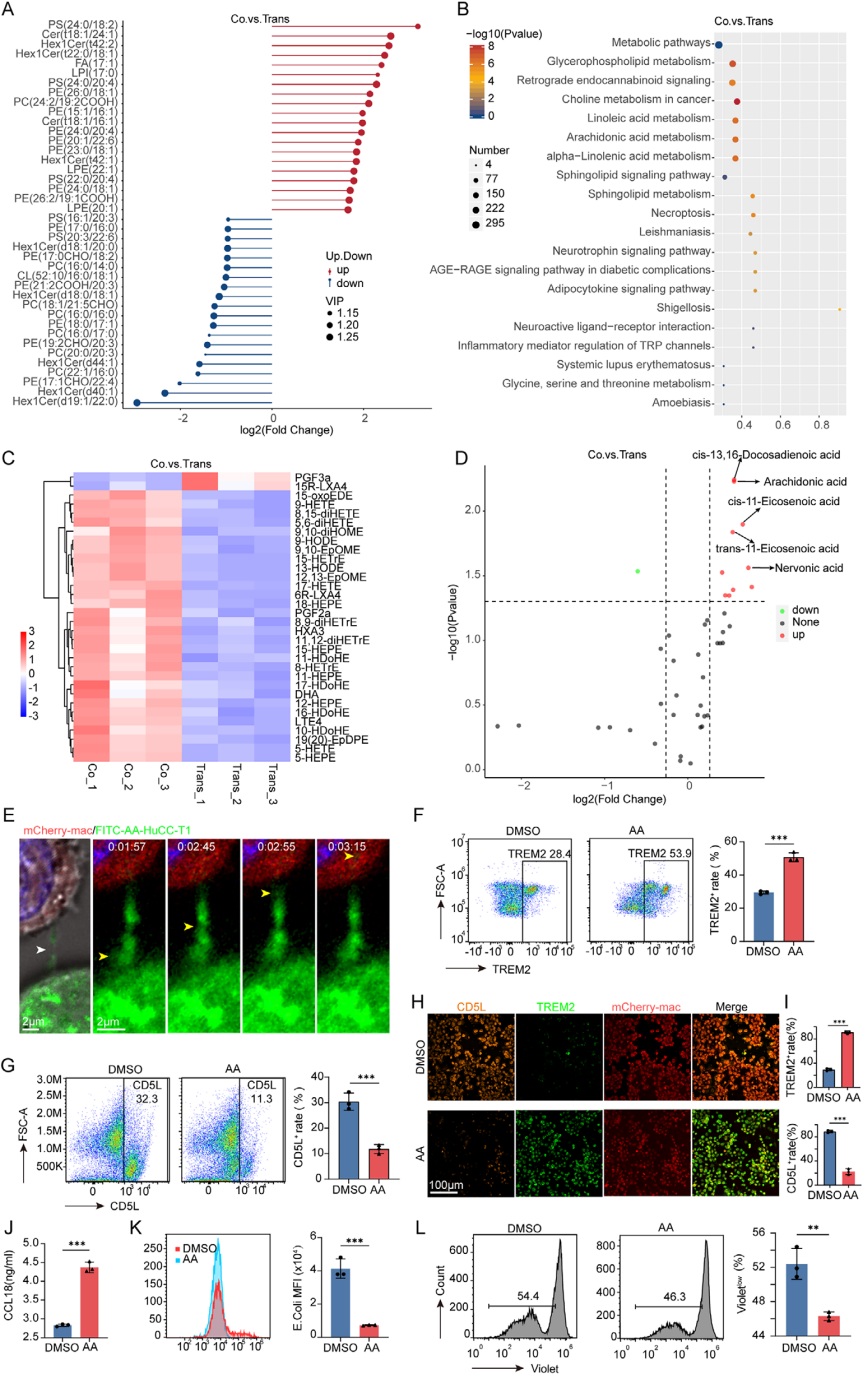
TNT‐mediated AA transfer drives macrophage phenotypic and functional reprogramming. A) Lipidomics profiling reveals distinct lipid compositions in macrophages from Coculture (Co) versus Transwell (Trans) systems. B) KEGG pathway enrichment analysis of upregulated lipid species in Coculture macrophages. C,D) Specific elevation of oxidized lipids (C) and fatty acids (D), including AA, in Coculture macrophages. E) Time‐lapse imaging of FITC‐AA‐labeled HuCC‐T1 cells (green) transferring AA to mCherry^+^ macrophages (red) via TNTs (white arrows). Yellow arrows in *t*‐axis panels (right) track AA transport dynamics. F–I) AA‐induced macrophage reprogramming: (F‐G) Flow cytometry quantification of TREM2 (F) and CD5L (G) expression in AA‐treated versus DMSO‐control macrophages. (H‐I) Immunofluorescence validation of TREM2 and CD5L positivity rates in DMSO and AA groups. J) Increased CCL18 secretion in AA‐treated macrophages. (mean ± SEM; n = 3). K,L) Functional consequences of AA transfer: (K) Flow cytometry analysis of macrophage phagocytic function on *E. coli* in AA‐treated versus DMSO‐control macrophages. (L) Proliferation of CD8^+^ T cells co‐cultured with macrophages. Data are mean ± SD; n = 3. Significance levels: ^*^ *p* < 0.05, ^**^ *p* < 0.01, ^***^ *p* < 0.001. AA, arachidonic acid; Co, Coculture; Trans, Transwell.

Lipidomics analysis revealed that upregulated lipid molecules in the Coculture group macrophages tended to associate with unsaturated fatty acid chains, whereas downregulated lipids were primarily linked to short‐chain or saturated fatty acids (**Figure** **7**A; Table S1, Supporting Information). Notably, significantly upregulated lipid species, such as PS (24:0/20:4) and PE (24:0/20:4), contained 20:4 fatty acid chains, likely corresponding to AA. KEGG pathway analysis further demonstrated that these upregulated lipids were significantly enriched in AA and linoleic acid metabolic pathways (Figure 7B). Additionally, oxidized lipid products significantly elevated in the Coculture group included AA oxidation derivatives (e.g., 5‐HETE, 17‐HETE, and 9‐HETE)^[^ ^31^ ^]^ and oxidation products derived from other unsaturated fatty acids (e.g., HEPE, HDoHE, and HETrE)^[^ ^32^, ^33^, ^34^ ^]^ (Figure 7C; Table S2, Supporting Information). Targeted metabolic profiling identified AA and other long‐chain unsaturated fatty acids, such as cis‐13,16‐docosadienoic acid and cis‐11‐eicosenoic acid, as significantly upregulated in macrophages from the Coculture group (Figure 7D; TableS3, Supporting Information). Collectively, these results suggest that long‐chain unsaturated fatty acids, primarily AA, are the major fatty acids transferred via TNTs.

To further validate this process, HuCC‐T1 cells were treated with exogenous FITC‐labeled AA and co‐cultured with macrophages. Observations demonstrate that FITC‐labeled AA is transported from HuCC‐T1 cells to macrophages via TNTs, as illustrated in Figure 7E. These findings provide compelling visual evidence of an intercellular lipid exchange pathway facilitated by TNTs.

To explore the functional consequences of TNTs‐mediated AA transfer, exogenous AA was added to macrophages. This treatment significantly increased TREM2 expression and decreased CD5L expression in macrophages (*p* < 0.05, Figure 7F,G). Immunofluorescence analysis further confirmed that macrophages receiving AA exhibited a higher percentage of TREM2 positivity and a lower percentage of CD5L positivity compared to the DMSO control group, with statistically significant differences (Figure 7H,I), indicating a phenotypic polarization toward the TREM2⁺ phenotype. Furthermore, macrophages treated with AA exhibited significantly increased secretion of the pro‐tumorigenic chemokine CCL18 (*p* < 0.05, Figure 7G).

Functional assays demonstrated that exogenous AA treatment significantly impaired macrophage phagocytic activity, as evidenced by reduced *E. coli* phagocytosis in flow cytometry analysis (*p* < 0.05, Figure 7K) and confirmed by confocal microscopy using Mander's coefficient (Figure S5A, Supporting Information). Additionally, AA treatment compromised phagocytosis of tumor cells (Figure S5B, Supporting Information) and markedly suppressed CD8⁺ T cell proliferation (*p* < 0.05, Figure 7L).

These results indicate that AA‐driven macrophage reprogramming weakens their antitumor immune responses, thereby reinforcing their tumor‐promoting functions.

In conclusion, our findings demonstrate that TNTs‐mediated AA transfer plays a pivotal role in driving macrophage polarization toward the TREM2⁺ phenotype and reprogramming their functional properties.

### 2.9 TNTs‐Mediated Material Transfer Activates the PI3K‐AKT Signaling Pathway and Regulates Macrophage Phenotype and Function

**Figure 8:**
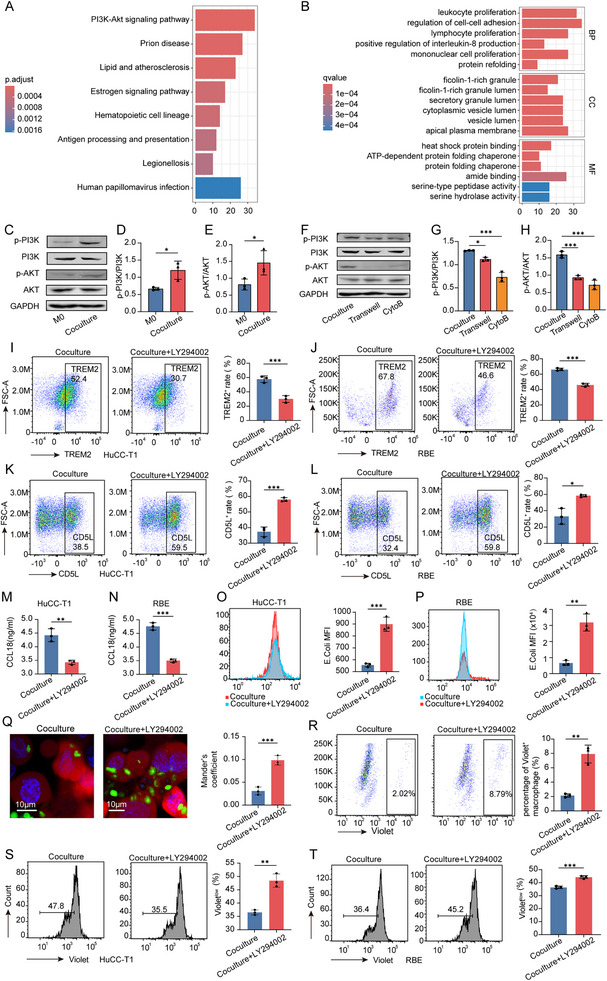
TNT‐mediated material transfer activates PI3K‐AKT signaling to modulate macrophage phenotype and function (A‐B) Pathway analysis of Coculture‐educated macrophages: A) KEGG enrichment of upregulated signaling pathways. B) Gene Ontology (GO) functional annotation of differentially expressed genes. (C‐H) PI3K‐AKT pathway activation: C) Western blot analysis of p‐PI3K and p‐AKT in M0 versus Coculture macrophages. D,E) Quantification of p‐PI3K/PI3K (D) and p‐AKT/AKT (E) ratios. Data are mean ± SD; n = 3. F–H) Reduced PI3K‐AKT activation in Transwell and CytoB‐treated groups compared to Coculture controls. Data are mean ± SD; n = 3. I–N) Phenotypic marker modulation by PI3K inhibition: TREM2 (I‐J), CD5L (K‐L), and CCL18 (M‐N) expression in Coculture macrophages treated with LY294002 (PI3K inhibitor) versus untreated controls, validated in HuCC‐T1 and RBE systems. Data are mean ± SD; n = 3. (O‐T) Functional consequences of PI3K‐AKT signaling: O,P) Flow cytometric quantification of macrophage phagocytic function against *E. coli* in HuCC‐T1 (O) and RBE (P) system. Q) Confocal microscopy analysis of macrophage phagocytic function against *E. coli* (Mander's coefficient). R) Assessment of macrophage phagocytic function against tumor cells. S,T) Proliferation of CD8^+^ T cells in HuCC‐T1 system (S) and RBE system (T). Data are mean ± SD; n = 3. Significance levels: ^*^ *p* < 0.05, ^**^ *p* < 0.01, ^***^ *p* < 0.001.

To explore the mechanisms by which tumor cell‐driven TNTs formation affects macrophages, we performed comparative transcriptome analysis on macrophages in the Coculture and Transwell systems (Table S4, Supporting Information). Transcriptome analysis revealed significant enrichment of pathways associated with tumor growth and lipid metabolism, including the PI3K‐AKT signaling pathway and pathways related to lipid metabolism and atherosclerosis (**Figure** **8**A).^[^ ^35^ ^]^ GO functional analysis further indicated enrichment of biological processes critical for tumor initiation and progression, such as the positive regulation of IL‐8 production and regulation of intercellular adhesion (Figure 8B).^[^ ^36^, ^37^ ^]^ These findings suggest that TNTs formation significantly alters the gene expression profile of macrophages, particularly genes involved in tumor progression, immune regulation, and lipid metabolism.

Specifically, genes upregulated in macrophages from the Coculture group were significantly enriched in the PI3K‐AKT signaling pathway, indicating that TNTs‐mediated material transfer might activate this pathway (Figure 8A). This hypothesis was validated by western blot analysis, which showed a marked increase in the p‐AKT/AKT and p‐PI3K/PI3K ratios in macrophages after Coculture compared to baseline levels (Figure 8C–E). Furthermore, macrophages in the Coculture group exhibited significantly higher activation of the PI3K‐AKT pathway compared to the Transwell and CytoB groups (Figure 8F–H), confirming that TNTs‐mediated material transfer induces PI3K‐AKT signaling activation.

To further elucidate the role of the PI3K‐AKT pathway in macrophage phenotypic transformation and functional reprogramming, we added the PI3K‐AKT pathway inhibitor LY294002 to the Coculture system. Inhibition of this pathway significantly decreased TREM2 expression (Figure 8I,J) and increased CD5L expression (Figure 8K,L). Additionally, the secretion of the pro‐tumorigenic chemokine CCL18 was markedly reduced (Figure 8M,N). Inhibition of the PI3K‐AKT pathway partially restores macrophage phagocytic capacity, as demonstrated by increased phagocytosis of *E. coli* in flow cytometry (Figure 8O,P) and confocal microscopy with Mander's coefficient (Figure 8Q), alongside enhanced phagocytosis of tumor cells (Figure 8R). This indicates that the pathway suppresses macrophage function critical for clearing tumor cells, and its inhibition alleviates this suppression. Furthermore, CD8⁺ T cell proliferation is partially recovered (Figure 8S,T), suggesting that PI3K‐AKT signaling contributes to immune suppression by limiting cytotoxic T cell activity, with its blockade enhancing anti‐tumor immunity. These results indicate that PI3K‐AKT signaling activation is a critical mediator of TNTs‐driven macrophage reprogramming.

In conclusion, our findings demonstrate that TNTs‐mediated material transfer activates the PI3K‐AKT signaling pathway in macrophages, driving their phenotypic transformation toward the TREM2⁺ pro‐tumorigenic phenotype and functional reprogramming. This activation not only modulates macrophage phenotype and function but also fosters an immunosuppressive microenvironment, thereby promoting tumor progression.

### 2.10 AA Activates the PI3K‐AKT Signaling Pathway and Regulates Macrophage Phenotype and Function

As previously described, TNTs‐mediated fatty acids transfer mainly involves AA and other long‐chain fatty acids, with transcriptomic analysis identifying PI3K‐AKT signaling pathway activation as a downstream effect of this transfer. To determine whether AA directly activates this pathway and regulates macrophage phenotypic and functional changes, we conducted a series of experiments.

**Figure 9:**
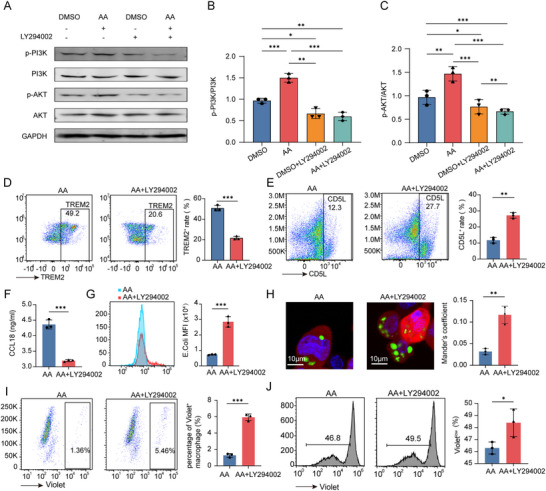
AA activates PI3K‐AKT signaling to modulate macrophage phenotype and function. A–C) PI3K‐AKT pathway activation by AA: (A) Western blot analysis of p‐PI3K and p‐AKT in macrophages treated with AA or AA + LY294002 (PI3K inhibitor). (B,C) Quantification of p‐PI3K/PI3K (B) and p‐AKT/AKT (C) ratios, demonstrating AA‐induced pathway activation and its suppression by LY294002. Data are mean ± SD; n = 3. D–F) Phenotypic marker regulation: Expression levels of TREM2 (D), CD5L (E), and CCL18 (F) in macrophages treated with AA or AA+ LY294002. Data are mean ± SD; n = 3. (G‐I) Functional impact of AA‐mediated signaling: G) Flow cytometric quantification of macrophage phagocytic function against *E. coli*. H) Confocal microscopy analysis of macrophage phagocytic function against *E. coli* (Mander's coefficient). I) Assessment of macrophage phagocytic function against tumor cells. Data are mean ± SD; n = 3. J) The ability to stimulate CD8^+^ T cell proliferation in macrophages treated with AA or AA+ LY294002. AA, arachidonic acid. Significance levels: ^*^, *p* < 0.05; ^**^, *p* < 0.01; ^***^, *p* < 0.001.

Western blot analysis demonstrated that treatment with exogenous AA significantly increased the p‐AKT/AKT and p‐PI3K/PI3K ratios in macrophages, indicating activation of the PI3K‐AKT signaling pathway (*p* < 0.05, **Figure** **9**A–C). Conversely, adding the pathway inhibitor LY294002 significantly reduced these ratios, confirming that the pathway was effectively suppressed (*p* < 0.05, Figure 9A–C). These results confirm that AA can activate the PI3K‐AKT signaling pathway, suggesting that TNTs‐mediated AA transfer may regulate macrophage function through this mechanism.

To validate whether AA‐induced macrophage phenotypic and functional changes depend on the PI3K‐AKT pathway, we co‐treated macrophages with exogenous AA and LY294002. Flow cytometry revealed that, compared to AA treatment alone, AA+ LY294002 treatment significantly reduced TREM2 expression (Figure 9D), increased CD5L expression (Figure 9E), and decreased CCL18 secretion (*p* < 0.05, Figure 9F). These findings indicate that inhibiting the PI3K‐AKT pathway reverses AA‐induced macrophage polarization. Functional assays demonstrated that macrophages in the AA + LY294002 group exhibited significantly enhanced phagocytic activity. This was evidenced by increased phagocytosis of *E. coli*, as confirmed through flow cytometry analysis (Figure 9G) and laser confocal microscopy using Mander's coefficient (Figure 9H). Additionally, the group showed improved phagocytosis of tumor cells (Figure 9I). Furthermore, a statistically significant improvement in CD8⁺ T cell proliferation was observed in the AA + LY294002 group compared to the AA treatment alone (P < 0.05, Figure 9J). This suggests that blocking the PI3K‐AKT pathway partially restores antitumor immune functions, counteracting AA‐mediated functional reprogramming.

In summary, our findings demonstrate that AA induces macrophage phenotypic transformation and functional reprogramming by activating the PI3K‐AKT signaling pathway. Inhibiting this pathway partially reverses these changes, offering novel insights into the metabolic regulation of macrophages within the TME and identifying potential therapeutic targets.

## 3 Discussion

This study is the first to confirm that in iCCA, tumor cells transfer long‐chain fatty acids, primarily AA, to macrophages via TNTs. This process drives macrophage polarization toward a pro‐tumorigenic TREM2⁺ phenotype while suppressing macrophage phagocytic function and reducing their ability to stimulate CD8⁺ T cell proliferation. Further investigation revealed that TNTs‐mediated AA transfer significantly activates the PI3K‐AKT signaling pathway, a key mechanism in macrophage phenotypic transformation and functional reprogramming. Inhibition of this pathway partially reversed the immune‐suppressive phenotype, restored macrophage function, and enhanced anti‐tumor immune responses. These findings provide novel mechanistic insights into TNTs‐mediated tumor‐immune cell interactions and identify TNTs and the PI3K‐AKT pathway as promising therapeutic targets for modulating the TME.

While cytokines, exosomes, and microvesicles have been extensively studied as mediators of intercellular communication, their diffusion within the dynamic 3D tumor stroma often limits the specificity and efficiency of signaling.^[^ ^4^, ^6^ ^]^ In contrast, TNTs serve as direct conduits for the targeted transfer of molecules, providing a deterministic and efficient communication route. Previous studies have highlighted TNTs’ role in promoting cancer cell metabolic plasticity, invasiveness, and therapy resistance through the transport of RNA, proteins, and organelles.^[^ ^20^ ^]^ However, research on TNTs in iCCA is sparse, and this study is the first to demonstrate their role in directly linking tumor cells and immune cells, thereby facilitating the formation of an immunosuppressive TME.

Macrophage phenotypes are shaped by their microenvironment, with M1 and M2 macrophages representing two extremes of activation. The anti‐inflammatory and pro‐tumorigenic effects of M2‐like macrophages are well‐documented but fail to capture the full complexity of macrophage activation states.^[^ ^38^ ^]^ In this study, we identified two distinct macrophage populations in iCCA: pro‐tumor TREM2⁺ macrophages and anti‐tumor CD5L⁺ macrophages, further clarifying the spectrum of macrophage phenotypic plasticity in TME remodeling. Consistent with prior findings in other cancers, AA and its oxidized derivatives were shown to promote TAMs polarization toward a pro‐tumoral phenotype.^[^ ^5^ ^]^ This study advances the field by demonstrating that TNTs‐mediated AA transport is a direct driver of macrophage functional reprogramming, emphasizing the interplay between lipid transfer and immune regulation.

The PI3K‐AKT signaling pathway is a well‐established regulator of immune cell activation and macrophage polarization.^[^ ^39^ ^]^ Our study demonstrated that TNTs‐mediated AA transfer activates this pathway, inducing phenotypic transformation of macrophages toward a TREM2⁺ phenotype characterized by reduced phagocytic activity and impaired CD8⁺ T cell support. Importantly, pathway inhibition with LY294002 reversed these changes, restoring macrophage antitumor functions and reducing the secretion of pro‐tumorigenic chemokines such as CCL18. These findings not only confirm the central role of the PI3K‐AKT pathway in macrophage reprogramming but also highlight its potential as a therapeutic target for TME modulation.

TNTs are capable of transporting various substances, including lipids, proteins, RNA, and organelles, and play an important role in regulating the TME.^[^ ^20^ ^]^ Our study reveals that AA‐dominant long‐chain fatty acids, transported to macrophages via TNTs, may be key factors driving macrophage phenotype transformation. Increasing evidence suggests that TAMs adapt to their metabolic needs by enhancing lipid uptake or storage, which is closely linked to their immune‐suppressive functions.^[^ ^25^ ^]^ It has also been shown that tumor‐derived long‐chain fatty acids can enter macrophages via extracellular vesicles, thereby providing fuel for their pro‐tumoral activity.^[^ ^26^ ^]^ Our findings further confirm that fatty acids are transferred from tumor cells to macrophages through TNTs, representing one of the primary pathways for tumor‐derived lipid transfer. Although lipids may also be transported via vesicles, TNTs provide a more directed and purposeful mechanism for lipid delivery to macrophages. However, other molecules transported by TNTs, such as proteins, signaling molecules, and RNA, may work synergistically with lipids or independently influence macrophage function. We further validated the critical role of AA in macrophage phenotype transformation, demonstrating that exogenous AA can significantly induce macrophages to polarize toward a pro‐tumoral TREM2^+^ phenotype, inhibit their phagocytic function, and reduce their immune stimulatory effects on CD8^+^ T cells, even under conditions that do not rely on TNTs. In conclusion, although TNTs transport a variety of substances, AA, as a key lipid molecule, can independently trigger macrophage functional reprogramming. Future studies should explore how other components transported via TNTs may cooperate with AA to jointly regulate the dynamic changes in the TME.

In this study, after AA is transported to macrophages via TNTs, it significantly activates the PI3K‐AKT signaling pathway, driving functional reprogramming. The PI3K‐AKT signaling pathway has been widely demonstrated to play a key role in immune cell metabolic adaptation and phenotype transformation^[^ ^39^ ^]^ suggesting that the activation of this pathway by AA may be somewhat specific. However, AA may not be the only acting molecule, and whether it cooperates with other metabolic pathways to jointly drive the activation of the PI3K‐AKT pathway requires further investigation.

TNTs are not exclusive to iCCA; they are widely present in cancers such as breast, lung, and pancreatic cancers, primarily facilitating cancer cell‐to‐cell communication.^[^ ^20^ ^]^ This study is the first to highlight their role in tumor‐immune cell interactions in iCCA, raising the possibility that TNTs may mediate similar immunosuppressive effects in other cancers, such as hepatocellular carcinoma and pancreatic cancer. Future studies could investigate the role of TNTs in regulating other immune cells, including T cells and dendritic cells, providing a broader understanding of their impact on cancer immunotherapy.

This study primarily relied on in vitro coculture systems and nude mouse xenograft models, which do not fully capture the complexity of the human TME. Future validation using patient‐derived organoids or xenograft models could provide more clinically relevant insights. Additionally, while this study focused on AA, other molecules transported via TNTs, such as proteins, nucleic acids, and mitochondria, may independently or synergistically influence macrophage function and TME remodeling. Advanced techniques, including proteomics and single‐cell multi‐omics, could uncover the broader repertoire of TNTs‐transported molecules and their roles in immune regulation.

Moreover, the potential interactions between the PI3K‐AKT pathway and other regulatory pathways, such as mTOR and NF‐κB, remain unexplored. Investigating these interactions, as well as the effects of TME conditions such as hypoxia and acidic pH on TNTs formation, could yield additional insights into the regulation of immune cell behavior in tumors. Finally, constructing a TME cell communication network using live‐cell imaging and high‐resolution techniques could comprehensively reveal the dynamic interactions mediated by TNTs.

## 4 Conclusion

This study establishes TNTs as a core mechanism for material transfer between tumor cells and macrophages in iCCA, with AA acting as a critical cargo molecule that activates the PI3K‐AKT signaling pathway. By driving TAMs polarization and functional reprogramming, TNTs contribute to the immunosuppressive TME and promote tumor progression. Targeting TNTs formation and the PI3K‐AKT pathway represents a promising strategy for cancer immunotherapy, with potential applications across multiple tumor types.

## 5 Experimental Section

### ScRNA‐Seq Analysis

It was obtained scRNA‐seq data for iCCA (GSE138709) from the Gene Expression Omnibus (GEO) database (https://www.ncbi.nlm.nih.gov/geo/). Ethical approval was unnecessary as per the GEO guidelines. Dimensionality reduction was conducted, clustering, subclustering, and mitigating batch effects. Cell clustering was performed with a resolution of 0.8. Marker genes were identified for biliary epithelial cells, malignant cells, and macrophages based on previous studies.^[^ ^12^ ^]^ Subsequently, epithelial cells was extracted and analyzed marker genes (|logFC|> 1, adj.*p* < 0.05), followed by GO and KEGG enrichment analysis. Subpopulations of macrophages were identified and further clustered (resolution = 0.3), with two subpopulations showing significant distribution differences between the tumor and normal groups. Marker genes were identified for the two subpopulations (|logFC| > 0.25, adj.*p* < 0.05). R packages Seurat, Harmony, org.Hs.eg.db, and ggplot2 was utilized for analysis and visualization.

### Cell Lines

The iCCA cell line HuCC‐T1 was obtained from Pricella (Catalog No. CL‐0725, China). The iCCA cell line RBE and the human monocytic cell line THP‐1 were sourced from the Institute of Biochemistry and Cell Biology, Chinese Academy of Sciences (Shanghai, China). Both cell lines were cultured in 1640 medium (Gibco BRL), supplemented with 10% FBS (Gibco) and 1% penicillin‐streptomycin at 37 °C with 5% CO_2_. HIBEpiC cell line was sourced from ZQXZbio (Catalog No. ZQY009, China) and cultured in HIBEpiC‐Im medium (ZQXZbio, Catalog No. ZMY009).

THP‐1 cells were induced to differentiate into macrophages by treatment with 100 ng mL^−1^ phorbol‐12‐myristate‐13‐acetate (PMA, S1819, Beyotime) for 24 h. Subsequently, the cells were cultured in complete medium for an additional 24 h to mitigate the influence of PMA, resulting in the generation of M0.

### Patient Samples

Tissue sections were obtained from three iCCA cases and their adjacent normal tissues. The diagnoses were confirmed through histological and clinical examinations. None of the patients had undergone preoperative chemotherapy, radiotherapy, or immunotherapy. Written informed consent was obtained from all patients prior to sample collection. Ethical approval was granted by the Research Ethics Committee of the Second Hospital of Hebei Medical University (approval letter no. 2024‐R474).

### Transfection

Lentivirus transfection was conducted following previously established protocols.^[^ ^40^ ^]^ HuCC‐T1 cells were transfected with GFP negative control lentivirus (Genechem, China), while THP‐1 cells were transfected with mCherry negative control lentivirus (Genechem, China). Stably transfected cell lines were obtained by selection with 1ug mL^−1^ puromycin (BL528A, biosharp) for 7 days.

### Live Cell Microscopy

Cells from various treatment groups were transferred to a Live‐Cell Microscope Incubation System (TOKAI HIT) and observed under a laser confocal microscope at 37 °C with 5% CO₂.

### Cell Staining

For F‐actin staining, cells were fixed and permeabilized with Triton X‐100. The cells were then stained with Phalloidin AF488 (ShareBio, YP0059).

mCherry‐THP‐1 cells were induced to differentiate into mCherry‐M0 on gelatin‐coated coverslips and co‐cultured with logarithmically growing HuCC‐T1 cells. For DiO staining, HuCC‐T1 cells were labeled with DiO (C1993S, Beyotime), co‐cultured with mCherry‐M0 cells. Subsequently, cells were staining with Hoechst 33 342, and observed under a laser confocal microscope.

### Cell Staining—Live Cell Tracing Staining

CellTracker Green CMFDA (40721ES50) was purchased from Yeasen Biotech Co., Ltd. (Shanghai, China). Cells were stained with either CellTracker Green CMFDA at a final concentration of 1 µm for 30 min at 37 °C. Following staining, cells were washed twice to remove excess dye and subsequently cultured in fresh medium for further experiments.

For lipid staining, cells were stained using BODIPY 493/503 (D3922, Invitrogen) at 37 °C for 30 min. Following staining, further experiments were conducted.

For FITC‐AA staining, tumor cells were treated with FITC‐AA (R‐FT‐0020, Stargray) at 37 °C for 30 min. Following staining, further experiments were conducted.

### Treatment of Different Culture Systems—Coculture System

mCherry‐THP‐1 cells were seeded onto gelatin‐coated coverslips and induced to differentiate into mCherry‐M0. HuCC‐T1 or RBE cells were then seeded into the same culture dish at a 1:1 ratio and co‐cultured for 24 h.

### Treatment of Different Culture Systems—Transwell Assay

HuCC‐T1 or RBE cells were seeded into the upper chambers of a transwell system with 1 µm pores, while mCherry‐M0 were plated in the lower chambers. After 24 h of co‐culture, allowing free diffusion of molecules between the two chambers while preventing intercellular TNTs formation.

### Treatment of Different Culture Systems—CytoB Treatment

To inhibit the formation of TNTs, a known inhibitor of actin polymerization^[^ ^41^ ^]^ 350 nm CytoB was added to the co‐culture system (ab143482, Abcam). For the control group, an equivalent volume of DMSO, the solvent for CytoB, was added.

### Treatment of Different Culture Systems—AA Treatment of Macrophages

Macrophages were cultured in medium supplemented with 20 ng mL^−1^ AA (A3611, Sigma, United States) for 24 h, designated as the AA group. For the control group, an equivalent volume of DMSO, the solvent for AA, was added. Following incubation, the cells were collected for subsequent experiments.

### Treatment of Different Culture Systems—Cell Treatment With LY294002

The cells were stimulated with LY294002 (10 µm) for 6 h.

### Determination of Free Fatty Acids

Free fatty acids in the medium of the cocultured system and the top of the transwell chamber were determined using a nonesterified free fatty acid assay kit (A042‐2‐1, Nanjing Jiancheng Bioengineering Institute) following the manufacturer's instructions.

### Analysis of Fatty Acids Transfer Rate

HuCC‐T1 or RBE cells were stained with BODIPY 493/503 before being seeded in the culture system at a 1:1 ratio. Cells in Coculture and Transwell groups, as well as in DMSO and CytoB groups, were analyzed using flow cytometry and fluorescence microscopy. The percentage of BODIPY‐positive mCherry‐expressing cells was quantified using FlowJo software for flow cytometry data and ImageJ for fluorescence microscopy images.

### Analysis of Macrophage Lipid Accumulation

Cells from different treatment groups were stained with BODIPY 493/503 and analyzed using flow cytometry to measure the MFI of BODIPY in mCherry‐positive cells. Additionally, cells were co‐stained with BODIPY 493/503 and Hoechst 33 342, and the MFI of BODIPY in mCherry‐positive cells was evaluated in each group using laser confocal microscopy.

### Measurement of Macrophage Phenotype

Cells from each group, both treated and untreated, were prepared as single‐cell suspensions and stained with anti‐TREM2 (SC‐373828, Santa Cruz) and anti‐CD5L (SC‐390486, Santa Cruz), respectively. Flow cytometry analysis was then performed to determine the proportion of antibody‐positive cells among the mCherry‐positive population in each group.

### ELISA

The CCL18 protein level in the cell culture supernatant was measured using the CCL18 ELISA kit (RAB0051, Sigma–Aldrich, Germany) according to the manufacturer's protocol.

### Phagocytosis Assay

PHrodo Green‐labeled *E. coli* BioParticles (P35366, Thermo Fisher Scientific) were reconstituted in Hank's balanced salt solution with 5% FBS, and then added to the different treatment groups at a ratio of 1 vial per 2 × 10^6^ cells, following the manufacturer's instructions. After co‐incubation for 4 h, the cells were subjected to either flow cytometry or confocal laser scanning analysis. FlowJo or Image J software were utilized to assess and analyze the proportion of Green^+^ macrophages relative to the total macrophage population. Additionally, ImageJ was employed to calculate Mander's coefficient for colocalization analysis.^[^ ^42^ ^]^ HuCC‐T1 cells were labeled with CellTrace Violet (C34557, Thermo Fisher) following the manufacturer's protocol. Macrophages from different treatment groups were prepared as described previously. Tumor cells and macrophages were co‐cultured at a 2:1 ratio for 4 h in a humidified incubator at 37 °C with 5% CO_2_. Phagocytosis was assessed by flow cytometry. Data were analysed by calculating the percentage of Violet‐positive cells among macrophages using FlowJo software.

### Transwell Assay

HuCC‐T1 cells were collected and resuspended in serum‐free RPMI 1640 medium. A Corning Transwell system with 8‐µm pore inserts was assembled in a 6‐well plate. The lower compartment of each well was filled with RPMI 1640 medium supplemented with 10% FBS, while the upper compartment was loaded with an equivalent volume of serum‐free RPMI 1640 containing 10 000 HuCC‐T1 cells. To induce chemotaxis, recombinant CCL18 (4 ng mL^−1^) was introduced into the lower compartment. The setup was incubated for 20 h at 37 °C in a 5% CO₂ environment. Post‐incubation, the cells were fixed using methanol and stained with crystal violet. The number of cells that migrated to the lower side was determined by counting cells in three randomly chosen microscopic fields under a light microscope.

### CCK‐8 Counting Kit Assay

Cell proliferation was evaluated using the CCK‐8 assay in accordance with the manufacturer's guidelines. HuCC‐T1 cells were plated in a 96‐well plate at a density of 1000 cells per well. 10 µL of CCK‐8 reagent was introduced into each well, followed by a 2‐h incubation at 37 °C. Absorbance readings were subsequently obtained at 450 nm using a multi‐mode microplate reader. Data were normalized relative to the absorbance values of the control group for comparative analysis.

### Lipidomics (LC‐MS/MS)

Lipidomics analysis was performed using liquid chromatography‐tandem mass spectrometry. The experimental workflow included sample collection, lipid extraction, LC‐MS/MS detection, and data analysis. Differential compounds were selected based on the criteria of VIP>1.0, FC>1.5 or FC<0.667, and *p*‐value<0.05.

### Oxidized Lipids

Oxidized lipid species were detected using LC‐MS based on the high‐sensitivity SCIEX QTRAP 6500+ mass spectrometer platform. The threshold for selecting differential metabolites was set at a FC>1.5 or FC<0.667 with a *p*‐value<0.05. Hierarchical clustering analysis was performed on all differential metabolites between the comparison groups. The relative quantification values of the differential metabolites were normalized and clustered.

### Targeted Metabolomics

An ultra‐high performance liquid chromatography coupled with tandem mass spectrometry (UHPLC‐MS/MS) system (ExionLC AD UHPLC‐QTRAP 6500+, AB SCIEX Corp., Boston, MA, USA). Materials and Reagents: All 50 fatty acid standards were obtained from ZZ Standards Co., Ltd. (Shanghai, China). Isopropanol (Optima LC‐MS), acetonitrile (Optima LC‐MS), and formic acid (Optima LC‐MS) were purchased from Thermo Fisher Scientific (Fair Lawn, NJ, USA). Ultrapure water was obtained from Millipore (MA, USA). Metabolite Extraction: Metabolite extraction was performed using chromatographic and mass spectrometric methods. Differential metabolites were selected based on an FC threshold of > 1.2 or < 0.833, with a *p*‐value < 0.05.

### Macrophage Isolation from Coculture System

Macrophages were isolated using the EasySep Human CD11b Positive Selection and Depletion Kit (STEMCELL, Vancouver, Canada). Cells were dissociated using accutase. The cell suspension was centrifuged, and the pellet was resuspended in sorting buffer at a concentration of 1 × 10⁷ cells mL^−1^. CD11b antibody mix was added, followed by magnetic beads. After incubation, the sample was placed in an EasySep magnet for 3 min, and unbound tumor cells were discarded. The CD11b‐positive macrophages were then collected by resuspending the magnet‐bound cells in sorting buffer.

### Proliferation Assay of T Cells

CD8⁺ T cells were isolated from peripheral blood mononuclear cells (PBMCs) using the MojoSort Human CD8 Nanobeads Positive Selection Kit (BioLegend, 480 107). Briefly, PBMCs were separated from whole blood using density gradient centrifugation and washed with PBS. The cells were resuspended in MojoSort Buffer and incubated with 10 µL of MojoSort Human CD8 Nanobeads for 15 min on ice. After washing, cells were placed in the MojoSort magnet, and CD8⁺ T cells were isolated by removing the unbound fraction. The purified CD8⁺ T cells were resuspended in culture medium for further analysis. Macrophages and HuCC‐T1/RBE cells were separated using immunomagnetic bead isolation. The isolated CD8⁺ T cells were labeled with CellTrace Violet (C34557, Thermo Fisher) and added to each treatment group of macrophages at a 10:1 ratio (T cells: macrophages). The co‐culture was supplemented with 10 µg mL^−1^ anti‐human CD3 (AH1003110, PBM), 2 µg mL^−1^ anti‐human CD28 (AH1028110, PBM), and 50 ng mL^−1^ recombinant human IL‐2 (200‐02‐10, PeproTech). After 48 h, cells were collected, and T cell proliferation was assessed by CellTrace Violet dilution via flow cytometry.

### RNA Extraction, Library Construction, and Sequencing

Macrophages from both the Coculture and Transwell groups were collected, with cells from the Coculture group being separated using immunomagnetic bead isolation. Total RNA was extracted from three independent cell samples using TRIzol reagent (Aidlab, Beijing, China). RNA sequencing was performed by KaiTai‐Bio (Hangzhou, China). The integrity, concentration, and purity of the RNA samples were analyzed. cDNA was synthesized using the U‐mRNAseq Library Prep Kit (AT4221). The prepared samples were sequenced on an Illumina NovaSeq 6000 platform. Data cleaning involved removing reads containing adapter contamination, low‐quality reads, and reads with over 5% “N” bases using fastp (v0.21.0). Reads were aligned to the human genome (GRCh38) annotated in the Ensembl database (Homo_sapiens.GRCh38.101.gtf) using HISAT2 (version 2.2.1) with default parameters. Differentially expressed genes were identified using the limma package, with an adjusted P value <0.05 and logFC >1. Biological pathway analysis was conducted using the clusterProfiler package.

### Western Blot Analysis

Each treatment group of macrophages underwent lysis with RIPA buffer containing protease inhibitors. Following centrifugation, the protein concentration was determined using a Bradford assay, and ≈100 µg of protein was loaded onto 10% SDS‐PAGE gels and transferred onto PVDF membranes. After blocking, membranes were incubated with primary antibodies overnight at 4 °C, followed by fluorescence‐conjugated secondary antibodies (LI‐COR Biosciences). Protein bands were visualized using an Odyssey infrared imaging system and quantified with ImageJ software. The primary antibodies included anti‐PI3K (CY6951, 1:500, Abways), anti‐p‐PI3K (CY6427, 1:500, Abcam), anti‐AKT (CY5561, 1:500, Abcam), anti‐p‐AKT (CY5855, 1:500, Abcam), and anti‐GAPDH (1:5000, Abways), serving as an internal reference.

### The Animal Tumor Xenograft Model

A tumor xenograft model was established by subcutaneously injecting a 10:1 mixture of HuCC‐T1 cells and macrophages into the backs of 5‐week‐old BALB/c nude mice (20–25 g; HFK BIOSCIENCE, China). A total of 24 mice were divided into four groups: (1) Coculture group, where macrophages isolated from the co‐culture system were mixed with untreated HuCC‐T1 cells; (2) Transwell group, where macrophages isolated from the Transwell system were mixed with untreated HuCC‐T1 cells; (3) DMSO group, where macrophages isolated from the DMSO group were mixed with untreated HuCC‐T1 cells; and (4) CytoB group, where macrophages isolated from the CytoB group were mixed with untreated HuCC‐T1 cells.  Each group received 5 × 10⁶ tumor cells in a total volume of 100 µL via subcutaneous injection. Tumor volume and body weight were measured twice a week, with tumor volume calculated using the formula V = (a × b^2^)/2, where *a* and *b* represent the largest and smallest tumor diameters, respectively. Mice were sacrificed three weeks after tumor implantation. Throughout the experimental period, no significant body weight loss was observed in the mice. All animal experiments were approved by the Research Ethics Committee of the Second Hospital of Hebei Medical University (Approval No. 2024‐AE270) in accordance with laboratory animal welfare and ethical guidelines.

### H&E Staining

First, deparaffinize the tissue sections and rehydrate them through a graded ethanol series. Then, stain the cell nuclei with hematoxylin and the cytoplasm with eosin. Finally, dehydrate, clear, and mount the sections for microscopic examination.

### Immunofluorescence/Multiple Immunofluorescence

Tissue sections were sequentially treated with endogenous peroxidase blocking, permeabilization, and BSA blocking. The sections were then incubated overnight at 4 °C with primary antibodies: anti‐TREM2 (ER1918‐04, 1:500, huabio), anti‐CD5L (17224‐1‐AP, 1:500, Proteintech), anti‐CD68 (PA6293, 1:500, Elabscience) and anti‐α‐tubulin (ab179484, 1:500, abcam). Afterward, sections were incubated with HRP‐conjugated secondary antibodies. Signal amplification was performed using the TSA Kit (SB‐Y6112, ShareBio), followed by antibody elution with elution buffer at 37 °C. The sections were then mounted using an anti‐fade reagent. Imaging and data analysis were carried out using a laser scanning confocal microscope and ImageJ software.

### Statistical Methods

Data were analyzed using R software (version 4.2.2) with appropriate packages and statistical methods. ImageJ and FlowJo software were used for analysis. SPSS (version 26) was utilized for further statistical analysis, and GraphPad Prism (version 9.5.1) was employed for data visualization. Statistical comparisons between the two groups were conducted using unpaired two‐tailed heteroscedastic *t‐*tests. Multiple groups were compared using one‐way ANOVA tests with Tukey's post‐hoc correction for multiple comparisons. In each experiment, ≈100 cells were randomly selected and analyzed under a confocal microscope to count the number of TNTs and determine the percentage of cells connected by them. Experiments were performed in triplicate. Each experiment included at least three biological replicates. A significance level of *p* < 0.05 was considered statistically significant, denoted as ^*^ *p* < 0.05, ^**^ *p* < 0.01, and ^***^ *p* < 0.001.

### Abbreviations

iCCA, intrahepatic cholangiocarcinoma; TAMs, tumor‐associated macrophages; TNTs, tunneling nanotubes; AA, arachidonic acid; TME, tumor microenvironment; scRNA‐seq, single‐cell RNA sequencing; HIBEpiCs, human intrahepatic biliary epithelial cells; MIF, multiple immunofluorescence; rCCL18, recombinant CCL18; CCK‐8, Cell counting kit‐8; TIME, tumor immune microenvironment; ELISA, enzyme‐linked immunosorbent assay; GEO, Gene Expression Omnibus; PMA, phorbol‐12‐myristate‐13‐acetate; CytoB, Cytochalasin B; MFI, mean fluorescence intensity; FC, fold change; PBMCs, peripheral blood mononuclear cells; H&E staining, hematoxylin and eosin staining.

### Ethics Approval

The animal study was approved by the Research Ethics Committee of the Second Hospital of Hebei Medical University (Approval No. 2024‐AE270). The study also received approval from the Research Ethics Committee of the same institution (Approval No. 2024‐R474) for the research involving human samples. Written informed consent was obtained from all patients prior to sample collection.

## Conflict of Interest

The authors declare no conflict of interest.

## Supporting information



Supporting Information

Supplemental Video 1

Supplemental Video 2

Supplemental Video 3

Supplemental Video 4

Supplemental Table 1

Supplemental Table 2

Supplemental Table 3

Supplemental Table 4

## Data Availability

The data that support the findings of this study are available in the supplementary material of this article.
