# Supplementary material for: Role of Tunneling Nanotubes in Arachidonic Acid Transfer and Macrophage Function Reprogramming in Intrahepatic Cholangiocarcinoma
Source: Adv Sci (Weinh). 2025 Jun 24;12(35):e00148. doi: 10.1002/advs.202500148 (PMC12462918; doi:10.1002/advs.202500148)
Supplement: Supplementary file 1 — Supporting Information [file ADVS-12-e00148-s004.docx]

Supporting Information

**Role of Tunneling Nanotubes in Arachidonic Acid Transfer and Macrophage Function Reprogramming in Intrahepatic Cholangiocarcinoma**

Meiru Chen^1,2^, Shangyumeng Zhao^3^, Xiaoli Xie^1^, Jiaqi Wang^1^, Miao Su^2^, Lixian Zhang^2^，Ruolin Cui^1^, Dongqiang Zhao^1^*

^1^ Department of Gastroenterology, The Second Hospital of Hebei Medical University, Hebei Key Laboratory of Gastroenterology, Hebei Institute of Gastroenterology, Hebei Clinical Research Center for Digestive Diseases, Shijiazhuang, 050000 Hebei Province, China

^2^ Department of Gastroenterology, Hengshui People’s Hospital, Hengshui, 053000 Hebei Province, China

^3^ Department of Preventive Medicine, College of Public Health, Hebei Medical University, Shijiazhuang, China.

*Corresponding author: Dongqiang Zhao; Department of Gastroenterology, The Second Hospital of Hebei Medical University, Hebei Key Laboratory of Gastroenterology, Hebei Institute of Gastroenterology, Hebei Clinical Research Center for Digestive Diseases, Shijiazhuang, Hebei Province, 050000, China

Email: [zhao_dongqiang@hebmu.edu.cn](mailto:zhao_dongqiang@hebmu.edu.cn)


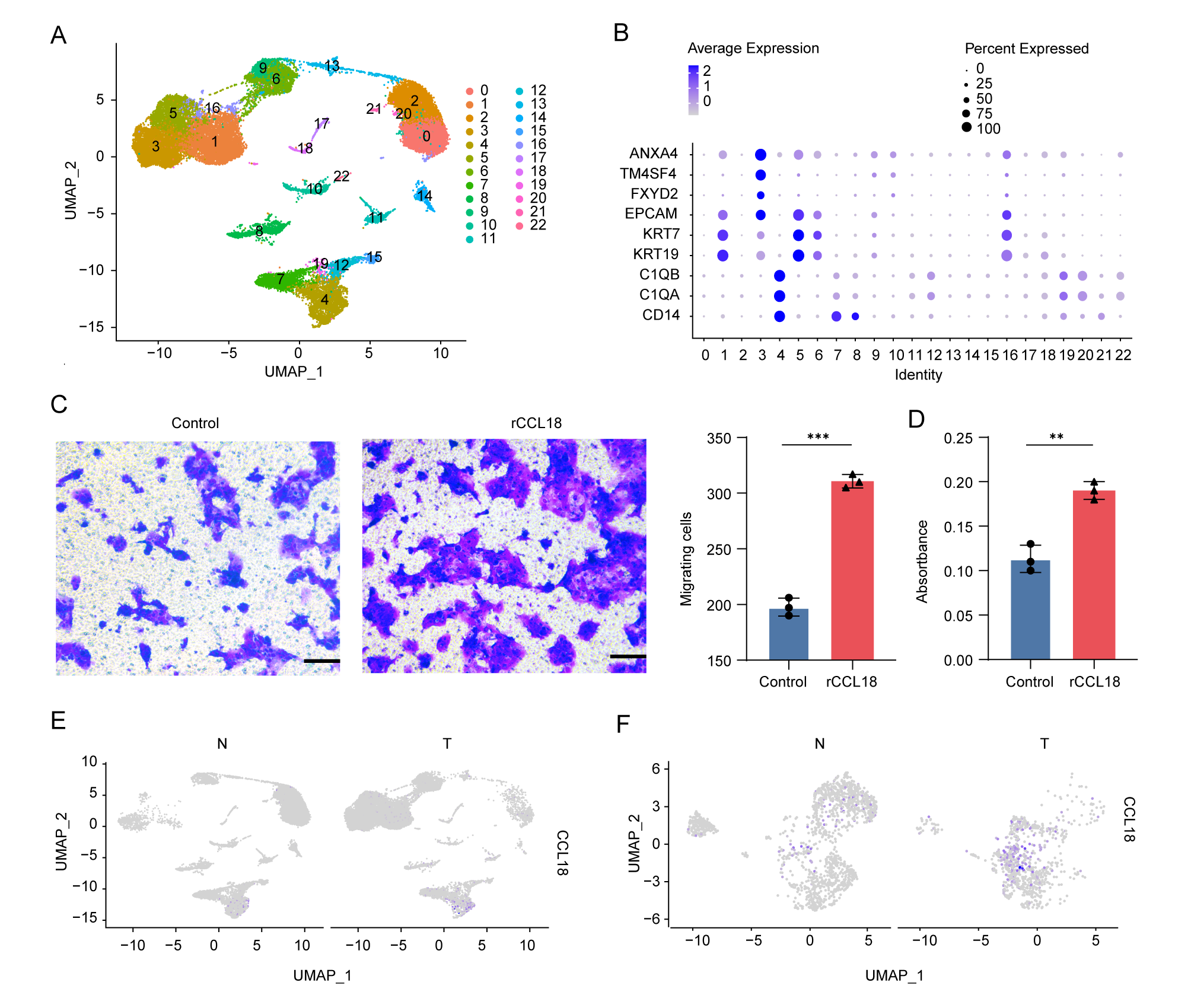


Figure S1: ScRNA-seq analysis. (A) UMAP plot displaying 23 clusters from scRNA-seq analysis. (B) The expression of marker genes for cholangiocytes and macrophages within each cluster. (C) A Transwell chamber was employed to evaluate the migratory activity of Hucc-T1 cells. Representative images are displayed on the left, while the statistical analysis of the migrated cell counts is presented on the right. Scale bar, 100 μm. Data are mean ± SD; n = 3. (D) Hucc-T1 cells were treated with recombinant CCL18 for 24 hours, and their proliferative activity was assessed using the CCK-8 assay in comparison to the untreated control group. Data are mean ± SD; n = 3. (E) The expression levels of CCL18 across cell types in tumor and normal tissues. (F) The expression levels of CCL18 within macrophage subtypes in tumor and normal tissues. rCCL18, recombinant CCL18; N, normal tissues; T, tumor tissues. Significance levels: ^**^*P*<0.01, ^***^*P*<0.001.


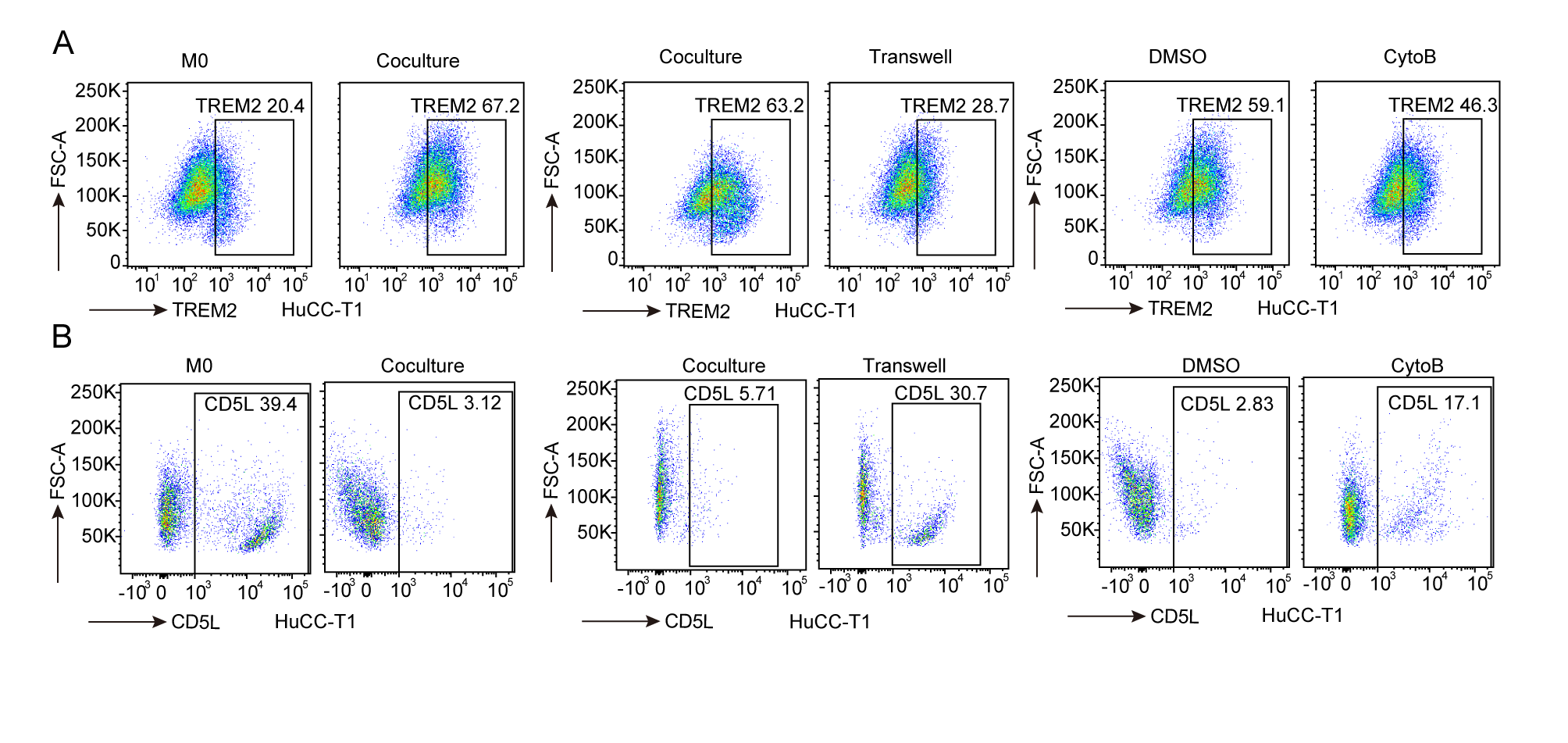


Figure S2: Effects of TNTs-mediated material transfer on macrophage phenotype. (A) Flow cytometric analysis of TREM2 expression across different groups in HuCC-T1 coculture system. (B) Flow cytometric analysis of CD5L expression across different groups in HuCC-T1 coculture system.


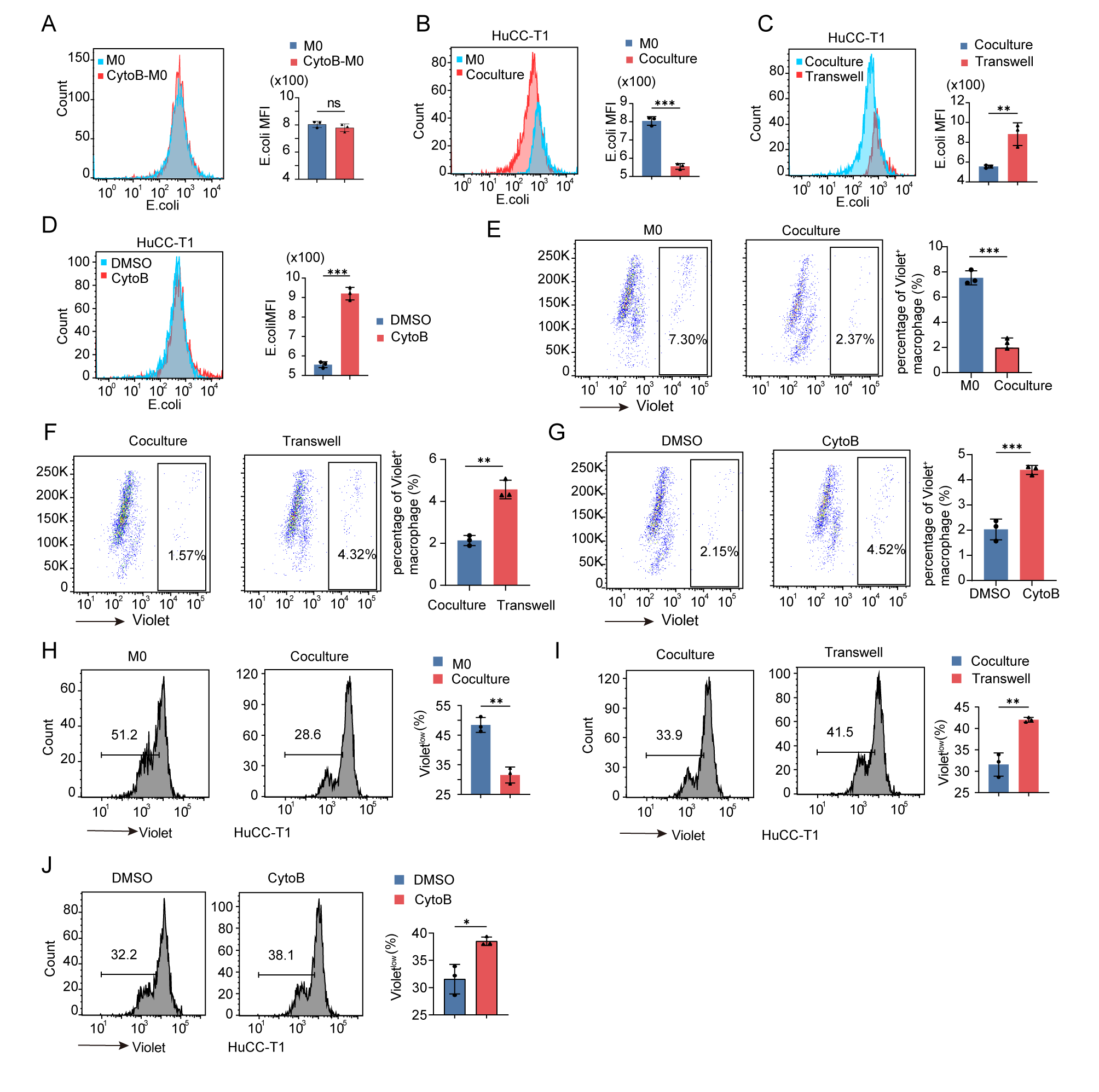


Figure S3: Functional consequences of TNT-mediated material transfer. (A) CytoB treatment did not significantly impair baseline macrophage phagocytic function. Data are mean ± SD; n = 3. (B-D) Flow cytometry analysis of *E. coli* phagocytic activity comparing the Coculture group with M0, Transwell, and CytoB groups, respectively, along with statistical comparison. Results are shown for the HuCC-T1 coculture system. Data are mean ± SD; n = 3. (E-G) Assessment of macrophage phagocytic function on tumor cells, comparing the Coculture group with M0, Transwell, and CytoB groups, including statistical analysis. Data are mean ± SD; n = 3. (H-J) Proliferation of CD8^+^ T cells co-cultured with macrophages. Results are shown for the HuCC-T1 coculture system. Data are mean ± SD; n = 3. Significance levels: ^*^, *P*<0.05; ^**^*P*<0.01, ^***^*P*<0.001.


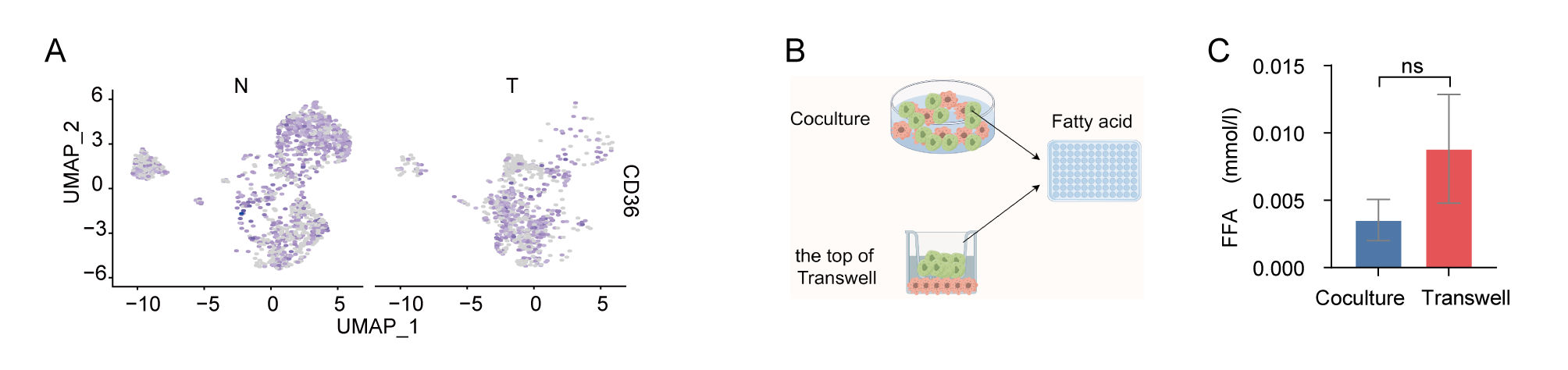


Figure S4: TNTs emerge as the primary approach for transferring tumor-derived fatty acids to macrophages. (A) Expression levels of CD36 in macrophage subtypes in tumor and adjacent tissues. (B) Flowchart of free fatty acid detection (by Figdraw). (C) Statistical comparison of free fatty acid concentrations between the Coculture group and the top chamber of the Transwell system. Data are mean ± SD; n = 3. N, normal tissues; T, tumor tissues. FFA, free fatty acid. ns, no significance.


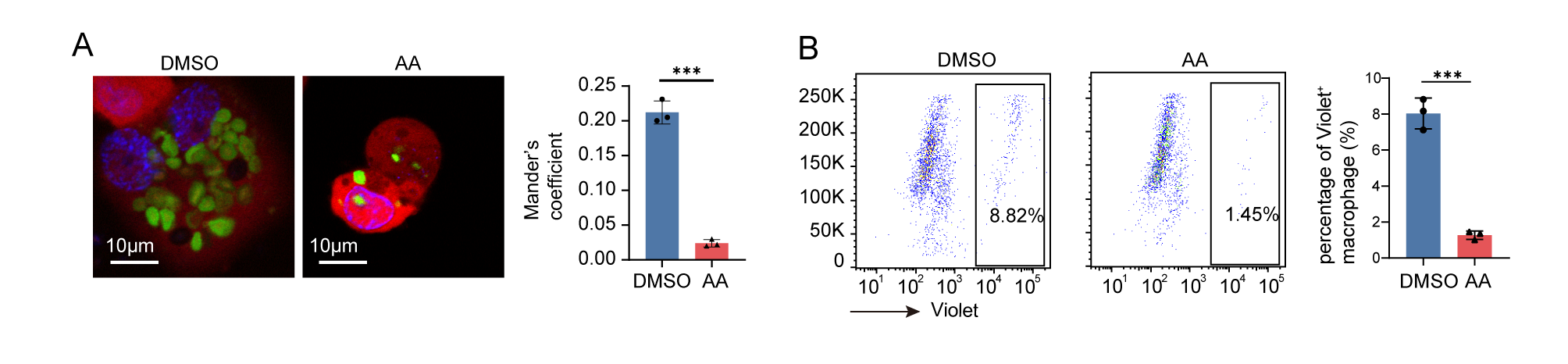


Figure S5: (A) Assessment of macrophage phagocytic function against *E. coli* in DMSO and AA treatment groups. (B) Assessment of macrophage phagocytic function against tumor cells in DMSO and AA treatment groups. Data are presented as mean ± SD; n=3. Significance levels: ^*^, *P*<0.05; ^**^*P*<0.01, ^***^*P*<0.001.

Suppl Video S1: Time-lapse confocal microscopy showing the formation of TNTs between DiO-labeled HuCC-T1 tumor cells and mCherry-labeled macrophages. The video captures the dynamic interaction and structural connection via TNTs over time. The video displays mCherry-labeled macrophages (red), DIO-labeled HuCC-T1 tumor cells (green), and Hoechst 33342-stained nuclei (blue).

Suppl Video S2: Time-lapse confocal microscopy demonstrating the transfer of DiO from HuCC-T1 tumor cells to mCherry-labeled macrophages through TNTs. The video highlights the movement of DiO along the TNTs from tumor cells to macrophages. The video displays mCherry-labeled macrophages (red), DIO-labeled HuCC-T1 tumor cells (green), and Hoechst 33342-stained nuclei (blue).

Suppl Video S3: Time-lapse confocal microscopy showing the transfer of BODIPY 493/503-labeled lipids from HuCC-T1 tumor cells to mCherry-labeled macrophages via TNTs. The video illustrates the lipid transfer process through the TNTs. The video displays mCherry-labeled macrophages (red), BODIPY 493/503-labeled HuCC-T1 tumor cells (green), and Hoechst 33342-stained nuclei (blue).

Suppl Video S4: Live-cell confocal microscopy over a 13-hour period with 10-minute intervals, revealing no instances of macrophages engulfing entire tumor cells. The video displays mCherry-labeled macrophages (red), GFP-labeled HuCC-T1 tumor cells (green), and Hoechst 33342-stained nuclei (blue).

Table S1: Lipidomics analysis of macrophages in Coculture and Transwell groups.

Table S2: Oxidized lipids of macrophages in Coculture and Transwell groups.

Table S3: Targeted Metabolomics of macrophages in Coculture and Transwell groups.

Table S4: Transcriptomic analysis of gene expression in macrophages in Coculture and Transwell groups.
